# Understanding the retention and support needs of UK first contact practitioner physiotherapists in primary care; a realist review

**DOI:** 10.1186/s12875-026-03197-6

**Published:** 2026-02-13

**Authors:** Rob Goodwin, Geoff Wong, Fiona Moffatt, Paul Hendrick, Richard Kelly, Pip Logan

**Affiliations:** 1 University of Nottingham, School of Medicine, Nottingham, UK; 2 Nuffield Department of Primary Care Health Sciences, University of Oxford, Oxford, UK; 3 University of Nottingham, School of Health Sciences, Nottingham, UK; 4 Nottingham CityCare Partnership CIC, Nottingham, UK; 5 Faculty of Health, Medicine and Behavioural Sciences, The University of Queensland, Brisbane, Australia

**Keywords:** First Contact Practitioner, Physiotherapy, Burnout, Primary Care, Turnover Intention, Realist Review, Workforce Sustainability, Role Clarity, NHS

## Abstract

**Background:**

First Contact Practitioner Physiotherapists (FCPPs) have been widely implemented in UK general practice through the Additional Roles Reimbursement Scheme to improve access to musculoskeletal (MSK) care and reduce GP workload. While evidence suggests clinical and economic benefits, concerns are emerging regarding practitioner burnout and turnover. Understanding how contextual factors contribute to these outcomes is essential for sustainable workforce planning.

**Aim:**

To explore how the UK based FCPP role interacts with workplace contexts to influence practitioner wellbeing, burnout, and turnover intentions, and to identify implications for support and service design.

**Methods:**

A realist review of UK-based literature examining the implementation and experiences of FCPPs and comparable primary care roles was conducted in line with RAMESES standards. Initial programme theories were developed through stakeholder consultation and iteratively refined using published evidence. Data were synthesised into context–mechanism–outcome configurations (CMOCs), organised across four domains: role characteristics, personal characteristics, caseload complexity, and working environment.

**Results:**

Thirty-three CMOCs were synthesised. Role ambiguity and poorly defined boundaries contributed to inappropriate referrals and role overload, intensifying an already complex caseload. These pressures were exacerbated by limited supervision and organisational support, leading to emotional strain, professional isolation, and increased burnout risk. Although practitioner experience and resilience could mitigate some effects, reliance on individual coping without adequate structural support undermined sustainability and contributed to turnover intentions.

**Conclusion:**

Burnout and turnover intentions among FCPPs arise primarily from systemic and organisational factors rather than individual shortcomings. Addressing role clarity, supervision, interprofessional integration, and workload alignment is essential to support practitioner wellbeing and ensure the long-term sustainability of FCPP roles.

**Trial registration:**

This review was registered with Prospero (CRD42024551933).

**Supplementary Information:**

The online version contains supplementary material available at 10.1186/s12875-026-03197-6.

## Background

The National Health Service (NHS) in the United Kingdom continues to face a mounting crisis in general practice, characterised by rising patient demand, an ageing population, and a sustained decline in the number of fully qualified general practitioners (GPs) [1]. Since 2015, there has been a 7% reduction in full-time equivalent GPs, a workforce shortfall that has coincided with significant increases in workload intensity and consultation volumes [2]. These pressures are particularly acute in socioeconomically deprived areas, where patients often experience multimorbidity and complex health needs [3].

In response, the NHS introduced the Additional Roles Reimbursement Scheme (ARRS) as part of the NHS Long Term Plan [4, 5]. The ARRS aims to diversify and expand the primary care workforce by integrating non-GP clinicians, including First Contact Practitioner Physiotherapists (FCPPs), into general practice teams [3–5]. The FCPP model allows patients with musculoskeletal (MSK) conditions to directly access experienced physiotherapists without requiring prior GP referral, thereby potentially improving access and reducing GP workload [6]. Since 2015 there has been a slowly growing body of evidence that suggests the FCPP services are clinically and economically effective and well received by all stakeholder groups [6–11].

The FCPP role is now broadly implemented across all four home-nations and has realised the aspiration to provide all adults in England with access to FCPP by 2024 [12]. This objective led to the rapid roll-out of the ARRS and, as a result, considerable heterogeneity in FCPP service model design and implementation [13, 14]. Consequently, there is emerging evidence that there are associated, if unanticipated, consequences of the FCPP role. Some of these were articulated early in the initiative [11, 15] and some potential resolutions sug­gested [16].

However, there is evidence to suggest that physiotherapists find that the specific nature of the novel FCPP role introduces new challenges for the workforce. Several features, including lack of awareness and understanding of the role [7, 17], the specificity of the primary care setting [14, 15] and the undifferentiated nature of the first contact encounter, described as adding uncertainty to the role [18–20], all appear to risk potential FCPP burnout [14, 21, 22]. Inadequate preparation for working autonomously in the primary care environment and limited access to structured mentorship or clinical governance frameworks have been suggested as contributing factors [18, 21]. Nozedar and O’Shea [22] reported that 13% of FCPPs met the threshold for burnout, with a further 16% at risk. Notably, 78% of FCPP clinicians were either exhausted or at risk of exhaustion.

The World Health Organisation (WHO included burnout in the 11th Revision of the International Classification of Diseases (ICD-11) as an occupational phenomenon [23]. Burnout in the broader healthcare professional workforce is associated with job dissatisfaction, reduced quality of care, and increased turnover intentions [24, 25]. Burnout in the wider physiotherapy workforce has been linked to increased absenteeism, decreased job satisfaction, reduced quality of care, and diminished professional efficiency [26, 27]. Although limited in the FCPP literature there is evidence to suggest that burnout could result in physiotherapists leaving the role [18]. Any risk of attrition of FCPPs is particularly concerning, given the strategic reliance on these roles to alleviate primary care pressures [28]. Retention challenges in the FCPP workforce could undermine the intended benefits of the model and exacerbate existing recruitment shortfalls across general practice [22]. Indeed, the NHS Long Term Workforce Plan aspires to support every general practice to have a musculoskeletal first contact practitioner (FCP) by 2032/33 and has staff retention as one of its three over-arching aims [28].

Turnover intention refers to the desire to relocate or leave an organisation to find a better job [29], and it is the most important indicator of actual leaving behaviour [30, 31]. A clear relationship between burnout and turnover intentions has been described [30–35]. Within the physiotherapy literature the evidence corroborates this relationship but is nascent [27]. This realist review explores the interrelationships between the evolving FCPP role, workplace challenges, burnout, and turnover intentions, against the background of ongoing primary care workforce constraints. By synthesizing current evidence, we aim to better understand how the implementation of the FCPP model is experienced by stakeholders and how these experiences may impact the long-term viability of the service. Specifically, it considers the implications of these dynamics for the retention and support needs of first contact practitioner physiotherapists in primary care. This review was registered with Prospero (CRD42024551933).

## Aims and objectives

By synthesizing current evidence, we aim to explore how workplace factors shape the implementation of the First Contact Practitioner Physiotherapist (FCPP) role and their impact on burnout, turnover intentions, and workforce sustainability, with the goal of informing FCPP support strategies.

The objectives are to:

1. Examine stakeholder experiences of implementing the FCPP role.
2. Explore how role demands, and workplace stressors contribute to burnout and attrition.
3. Assess implications for FCPP recruitment and retention.
4. Identify contextual factors affecting the sustainability of the FCPP model.
5. Inform recommendations about the support needs of FCPPs.in primary care.

## Methods

Realist synthesis was selected as an appropriate methodology for understanding the complex, context-dependent nature of this workforce transformation and service delivery model [36–38].

### Realist approach and philosophical orientation

Realist reviews are a theory-driven approach to synthesizing secondary data [39]. They are grounded in realist philosophy of science, which posits that outcomes are caused by mechanisms, which in turn are activated within certain contexts. Social programmes and policies (like ARRS) work by changing context [36, 37]. This approach moves beyond the question of "does it work?" to explore "what works, for whom, in what circumstances, how, and why?" [37, 38]. The causal explanations in realist reviews are expressed as context-mechanism-outcome configurations (CMOCs) (see Table 1 for definitions). In a realist review CMOCs are developed and tested (confirmed, refuted or refined) using secondary data and are embedded within a programme theory that describes how an intervention is thought to work and under what conditions [40].

**Table 1:** Conceptualizations of realist concepts [41]

Context Any condition that triggers and/or modifies the behaviour of a mechanism. Context refers to the important feature(s) of the circumstances in which an intervention ‘works’ (or a phenomenon happens) which ‘trigger’ the mechanisms that generate outcomes. Changes in context over time or in different settings will affect whether—and which—mechanisms are in operation
Mechanism The underlying process by which outcomes are caused. Mechanisms are usually descriptions of the tendencies, reasoning and behaviour of agents involved in a process or participants in an intervention and their response to the important context(s) in which they exist. Mechanisms are not the same as ‘variables or correlates’ that are associated with particular outcomes; instead, they offer an explanation for why and how observed outcomes happen
Programme theory A theory that describes what an intervention comprises and how it is expected to work, or the process by which the outcomes of interest are thought to come about (expressed as a narrative description or in a diagram). A realist programme theory is expressed in terms of the relationships between relevant context(s), mechanism(s) and outcome(s) (CMOCs)—and the relations between CMOCs
Context–mechanism–outcome (CMO) configuration ‘Context–mechanism–outcome configuration’; a diagrammatic or narrative description offering an explanation of the relationship between some particular context(s), mechanism(s) and outcome(s). Multiple CMOCs may exist within a single programme theory

Our realist review was conducted and reported in accordance with RAMESES (Realist And Meta-narrative Evidence Syntheses: Evolving Standards) quality and reporting guidelines [42]. The realist review followed the key stages of a realist review, as previously described by Pawson et al. [37, 42].

## Step 1: Developing the Initial Programme Theory/Theories (IPT/IPTs)

We began by developing our IPTs to understand the retention and support needs of FCPPs in primary care. We used our initial scoping of the literature and expert knowledge to develop our initial programme theories (Table 2). These rough IPTs were circulated to, and modified from feedback by, our stakeholder groups which included patients (n = 4) specialist MSK physiotherapists (n = 6) and senior members of the Chartered Society of Physiotherapy Practice and Development Team (n = 4). We attempted to focus on themes that were deemed relevant in understanding this complex intervention (implementation of the FCPP role and associated impact on burnout, turnover intentions, and workforce sustainability).

**Table 2:** Initial programme theories

Burnout
There appear to be several features of the FCPP role that could lead to physiotherapist burnout. This could be due to specific features of the FCPP role, such as the undifferentiated patients and the associated diagnostic/medical uncertainty, or it could be related to the systems and support available to the physiotherapists, such as overly busy clinics, access to resources/IT systems and supervision/support Physiotherapists describe several ‘responses’ to the FCPP role that could be associated with resultant burnout. These include lack of confidence, feeling overwhelmed, fear, dread, greater sense of responsibility, worry, deterioration in wellbeing, exhaustion The potential consequence is that physiotherapists describe an intention to reduce their hours working as an FCP or an intention to leave the role completely
Understanding There appears to be some confusion and lack of understanding of the FCP role. This lack of understanding appears in several stakeholders, including patients, general practice staff and GPs. The lack of understanding from stakeholders may add to the negative work experience of physiotherapists via several mechanisms
Embeddedness It appears that the embeddedness of the FCP service within a primary care setting/general practice is impactful. Interprofessional collaboration is a variable and impactful context in which the FCP role is situated. It appears that the more time the physiotherapist is present in the practice the greater the opportunities for collaboration- both formal (supervision and practice meetings) and informal. This appears to have several impacts including improved working relationships, improved understanding of the role and increased physiotherapist knowledge and confidence. This also is said to improve patient care
Capacity Where the capacity of FCP services is less than optimal (i.e. MSK demand exceeds FCP capacity) there may be several consequences. Waiting lists to see the FCP may develop, leading to frustration (staff & patient). This situation may be worsened when GPs utilise the FCP, MSK specialist resource for a ‘second opinion’. FCPs may, resultingly, streamline their assessment process in their adoption of a ‘GP approach’ and shorter assessment times. This may lead to a real, or perceived, increase in clinical risk, increased FCP stress and burnout, leading to turnover intentions FCP as a professional development opportunity Physiotherapists see the FCP role as a career development opportunity. However, some models of FCP implementation may result in the FCPs feeling under-prepared and ‘thrown in at the deep end’. They may experience a sense of isolation and subsequently this may add to their feelings of being less well equipped/able to cope- particularly with medical/diagnostic uncertainty. If the model of FCP service and support means that their supervision is limited this adds to their negative experiences and leads to turnover intentions

## Step 2: Search strategy and evidence identification

### Formal search

The initial programme theory functioned as a springboard for our formal search for data. An information specialist helped in the development of a search strategy. Searches were run on the following databases: MEDLINE (Ovid), CINAHL Plus, Embase, PsycINFO during the period 2000-June 2024.

Search terms brought together the three overarching themes of professional groups included in ARRS, location (primary care/general practice) and anticipated/related psychological impacts of these new developing roles. (see Additional File 1 for the full strategy, MEDLINE).

## Step 3: Selection and appraisal of documents

Screening was conducted in two stages:

i) Title and Abstract Screening: Inclusion criteria included data related to NHS settings, primary care, general practice, health care professional and non-professional groups and exclusion criteria included data related to physicians/GPs/Nurses, non-NHS settings, on-English publications, related specifically to Covid-19. All references were stored on Covidence systematic review software (www.covidence.org.). Initial screening was undertaken by RG, with 10–20% dual-screened (RG, RK) to ensure consistency. Any disagreements were resolved through discussion.
ii) Full text screening: All included full texts were reviewed for inclusion based on realist criteria of relevance and rigour [37, 43] and whether they were ‘fit for purpose’. We included all study designs (qualitative, quantitative, mixed methods), process evaluations, grey literature, case studies, and policy documents if they contained data relevant to developing or testing our theories and subsequent context, mechanism, outcome configurations (CMOCs). Our anticipation was that when a CMOC was based on a limited amount of data, we would look more closely at the methods used to make judgments about rigour. We assessed the explanatory power of the programme theory against criteria of consilience (accounting for as much of the data as possible), simplicity (not containing lots of caveats) and analogy (relating to what is already known) [43].

## Step 4: Data extraction and organisation

Full texts were imported into and coded within Covidence systematic review software (www.covidence.org.) and data were exported into an Excel file. Data extraction was undertaken by RG with 10% co-reviewed by RK. Characteristics of included full texts can be found in additional file 2. Coding employed both deductive (developing CMOCs from our IPT) and inductive (allowing new themes, contexts, mechanisms and outcome to be developed). Early coding focused on professional roles, role boundaries, individual characteristics, integration within primary care, psychological consequences related to the roles and issues pertaining to turnover intentions.

## Step 5: Data synthesis and theory refinement

Synthesis was guided by realist logic of analysis [37, 38]. CMOCs were iteratively developed by RG and circulated amongst the project group. Data were also used from the wider workforce literature on other ARRS roles to draw inferences that we judged were applicable to FCPP. Over the twelve months of the review the project team met every two months, where discussions took place. Within these group discussions a range of analytic processes were used to develop and test the CMOCs. These included juxtaposition (comparing explanations across professional groups, settings, and stakeholder groups), reconciliation (why something succeeded in some contexts but not others), and adjudication (giving greater weight to certain data sources e.g. those which were richer contextually or had greater explanatory insight). Once agreement was reached regarding CMOCs there was a process of consolidation, whereby multiple CMOCs were synthesized into overarching refined programme theories.

Throughout, retroductive and abductive reasoning was applied to infer underlying mechanisms from patterns in the data and to generate plausible explanations for observed outcomes [38].

### Stakeholder engagement

The initial stakeholder groups were consulted at the end of the synthesis process to help further refine the revised programme theories, to sense-check the findings and to enhance the practical relevance and credibility of the final programme theory.

Feedback from Patient and Public Involvement (PPI) contributors ensured that patient perspectives were adequately represented in the developing CMOCs.

### Reporting

This realist synthesis is reported following the RAMESES publication standards [38, 42]. A PRISMA diagram detailing the screening and selection process (additional file 3), a full list of included documents, (additional file 2) are provided in the supplementary materials.

### Findings

**Fig. 1:**
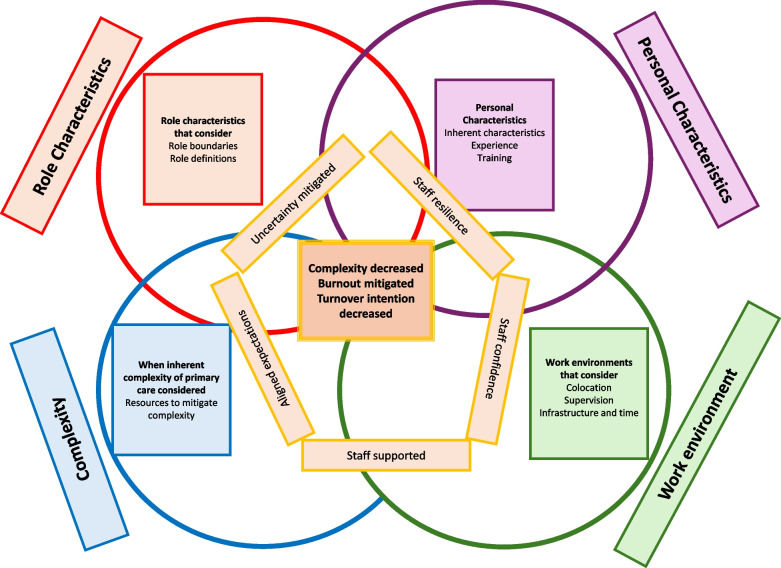
Proposed programme theory of how first contact practitioner physiotherapists can be supported in their role. In this figure, each over-arching theme is represented by a circle (role characteristics is red, personal characteristics is purple, complexity is blue and work environment is green). Supporting contextual considerations are identified in the boxes, within the circles. The outcomes of decreased complexity, burnout mitigation and decreased turnover intention are in the central box. The mechanisms resilience, aligned expectations, feeling supported, confidence and mitigated uncertainty (yellow rectangles) support these outcomes (adapted from [44])

Within this review the four overarching themes that were synthesized, with their sub-themes, are reported narratively with reference made to the underpinning 33 CMOCs. To support the reader several supplementary files are provided (Additional file 4, contains all the refined CMOCS, under theme headings and sub-themes, Additional file 5, full list of refined CMOCs and supporting sources, Additional file 6, full list of refined CMOCs and supporting data extracts). The refined programme theories and the overall, refined combined programme theory are reported in Table 3 and figuratively, in Fig. 1.

**Table 3:** Refined Programme Theories

Refined programme theories	
**Role Characteristics** The inherently complex nature of novel primary care roles such as First Contact Practitioner Physiotherapists (FCPPs), is intensified by unclear role definitions and poorly defined professional boundaries. This ambiguity leads to confusion among other practice staff and results in inappropriate patients and/or excessive numbers of patients with increased complexity and volumes that are beyond what was originally anticipated. Practitioners are often required to manage presentations outside the anticipated scope of their role, with professional boundaries overlapping. This can contribute to interprofessional tension and a lack of confidence in their own clinical decision-making. The uncertainty about what is expected of them, and the fear of overstepping professional limits, generates emotional distress and ethical concerns. Over time, this combination of clinical burden, professional insecurity, and systemic inefficiency can lead to burnout and increase the likelihood of practitioners leaving their roles	**Personal Characteristics** The personal characteristics of practitioners, such as resilience, optimism, empathy, and flexibility, play a vital role in enabling them to cope with the complexities and demands of their roles. However, while these traits can potentially mitigate burnout and support sustainability, over-reliance on them without structural support is problematic. Resilience is not purely innate but can be developed through exposure to challenging situations, opportunities for reflection and learning, and the confidence gained from setting clear professional boundaries. Training, especially when it addresses clinical uncertainty, complex patient presentations, and role-specific competencies, can enhance this resilience, yet is often insufficient. Similarly, relevant professional experience, especially when gained in diverse or complex clinical settings, is a key factor in building practitioners’ confidence, reducing anxiety, and improving their capacity to manage uncertainty
**Complexity** Caseload complexity is an inherent feature of novel primary care roles amplified by the clinical and social complexity of general practice populations and compounded by a lack of clarity in role definitions and poorly defined professional boundaries. This lack of clarity results in patients who are not appropriate for first contact practitioners in practitioners’ caseloads. It also leads to inappropriate expectations, leading to practitioners encountering patients with both complex multimorbidities and conditions that are beyond their scope of practice. The burden of responsibility, time pressures, and exposure to clinical uncertainty can undermine practitioners’ confidence, increase stress, and, in some cases, contribute to burnout and turnover intentions. While practitioners often adopt coping strategies to manage this complexity, such as seeking additional advice or spending more time with patients, these efforts can paradoxically increase workload and emotional strain	**Work Environment** The working environment plays a critical role in shaping the experience, performance, and sustainability of practitioners in novel primary care roles. Co-location within practice teams fosters informal communication, easier access to supervision, and interprofessional collaboration- factors which support practitioners’ confidence, clinical decision-making, and integration within teams. In contrast, remote or fragmented working models contribute to professional isolation, reduced access to peer and supervisory support, and a diminished sense of belonging. This isolation is exacerbated by modern working practices, inconsistent supervision structures, and limited opportunities for informal interaction. Service design factors- such as shorter appointment lengths, lack of administrative and non-clinical time, insufficient infrastructure, and unclear governance- compound the pressure practitioners face, leaving them professionally compromised and emotionally strained. The absence of ring-fenced resources for mentorship and the under-resourcing of alternative referral pathways further increases practitioner burden. When support structures such as supervision, peer networks, and protected time are absent or inconsistently applied, practitioners are left vulnerable to stress, burnout, and attrition
**Final Combined programme Theory** The sustainability and success of novel primary care roles such as First Contact Practitioner Physiotherapists (FCPPs) are fundamentally shaped by a complex interplay between role clarity, practitioner capacity, caseload complexity, and working environment. At the centre of this dynamic system is the alignment- or misalignment- between practitioner expectations, clinical demands, and the organisational structures designed to support them When roles are poorly defined, with unclear boundaries and expectations, this creates confusion among general practice staff, resulting in inappropriate referrals and an influx of patients whose needs exceed the intended scope of the role. This misalignment contributes to caseload complexity that overwhelms practitioners both clinically and emotionally, particularly when patients present with multimorbidity, socioeconomic challenges, or diagnostic uncertainty Practitioners draw on personal characteristics such as resilience, empathy, and adaptability to manage this complexity. However, these traits alone are insufficient mediators against the cumulative pressures of the role. Without adequate training, supervision, and role-appropriate preparation, even experienced clinicians report anxiety, reduced confidence, and moral distress- especially when they feel clinically compromised or unsupported The working environment acts as a crucial contextual moderator. Where FCPPs are co-located and embedded in multidisciplinary teams, they benefit from peer support, informal learning, and accessible supervision- factors that enhance resilience, confidence, and integration. Conversely, remote or fragmented deployment models contribute to professional isolation, a diminished sense of belonging, and fewer opportunities for real-time collaboration. These environmental deficits amplify the emotional and operational strain caused by unclear roles and complex caseloads Structural features of service design- such as shortened appointments, limited administrative time, and inadequate access to diagnostic tools- further restrict practitioners’ ability to manage complexity safely and effectively. These limitations not only increase stress and emotional fatigue but can also lead to ethical tension, perceived risk, and ultimately burnout and turnover intentions In sum, the FCPP role functions effectively when practitioners are provided with clear boundaries, integrated team environments, adequate supervision, protected learning time, and manageable caseloads. Without these structural and cultural supports, the cumulative effect of clinical burden, isolation, and professional insecurity places practitioners at risk of attrition, threatening the sustainability of these workforce innovations

## Theme 1; Role characteristics

### Theme 1a; Role boundaries, confusion, inappropriate referrals

It was clear that the roles included in the data were novel for each of the professional groups and, as such, by their nature, exposed practitioners to a level of clinical complexity that was both new and challenging (CMOC1).

*Participants highlighted that in their FCP role, they were dealing with more complex patients, which they had not anticipated at the outset: … ‘It’s the level of clinical complexity that you’re dealing with”. *[14]

However, beyond the inherent complex nature of the roles it was also evident that the roles varied widely and there was a lack of clarity in terms of role boundaries and role definitions (CMOC2-8).

There were several consequences, and significant forfeitures because of the lack of clear roles and role boundaries. General practice staff were confused about the remit of the roles, and this led to referral/signposting of inappropriate patients, outside of the professional scope of the practitioners. It could also lead to unrealistic expectations about the scope of practitioners. Both were described as further adding to practitioner caseload complexity and resulting in overwhelming the services with the creation of waiting lists. The lack of understanding regarding role and boundary was also seen, in the case of FCPPs, to result in patients being ‘referred’ by GPs for second opinions (not something that is meant to be within the remit of the FCPP), further overwhelming services. Unsurprisingly, this also impacted on the practitioners themselves with experiences of frustration, lack of confidence in providing care to patients who they felt sat outside their professional scope, high levels of role stress through workload overload and complexity (CMOC 4–6).

*[W]ith “their job scope and remit being poorly defined from the outset” and their role being “not well understood by external referrers”. This could lead to unrealistic expectations or confusion around what is achievable through social prescribing and to the referral of patients with complex needs that exceeded the remit of the link worker role *[45]

*A lack of role clarity existed, often leading to first contact physiotherapists acting as the second contact and the development of long waiting lists in some models, resulting in perceived inefficiencies that caused frustration *[21]

### Theme 1b; Boundaries, scope of practice

There also appeared to be some challenge in how practitioners assumed or rejected the additional responsibilities that were part of these advanced practice roles. For some, there was an enthusiasm to extend their scope, for others a feeling that they were ‘filling gaps’ [45] and some a sense that there was an expectation for them to do so. This ambiguity in terms of role boundaries and associated responsibilities was associated with a sense of increased risk to patients (CMOC 7, 8).

Once again, practitioners acknowledged the emotional burden that accompanied this lack of role boundary and definition clarity.

### Theme 1c; Role overlap, rivalries, tensions.

Significant role overlaps existed because unclear role boundaries and definitions often occurred, and this had the potential to create interprofessional tensions, which were detrimental to interprofessional working.

*Issues of interprofessional competition and professionals’ attempts to protect occupational jurisdiction in their work are well documented. Studies have revealed tensions between staff in relation to authority, legitimacy, expertise and efforts made to gain professional recognition in multidisciplinary working* [46]

## Theme 2; Personal characteristics

Resilience was reported in the data as an antidote to burnout [47]. Several individual characteristics such as optimism, flexibility, tolerance and empathy, were described as inherent in resilient practitioners [15, 17, 45, 47, 48]. Although important, it was suggested that these intrinsic features could not be solely relied on to sustain services.

*Clinician characteristics appear to be important…[g]ood communication skills, listening and clear provision of helpful information were noted as important to respondents, with further importance placed on personal attributes of FCPs, namely empathy, kindness, and friendly nature, making respondents feel comfortable and being respectfully treated* [17]

### Theme 2a; Exposure to stress, opportunities to learn, resilience

There appeared to be a complex relationship between exposure to stress, this exposure providing an opportunity for learning, the subsequent coping, and the development of resilience. Furthermore, this experience of stressful situations provided practitioners with the confidence to both articulate their own professional boundaries and work within them (CMOC 10–12).

#### Quote 1

*General exposure to, and experience of, different and challenging situations was a strong theme across groups, as this exchange illustrated:*

*‘Yes, difficult situations, good situations. But I think it’s the more challenging situations that builds up the resilience.’*

#### Quote 2

*Thus, having the confidence to be assertive was raised as a feature of resilience, in relation to understanding one’s role and professional identity, and when dealing with patients and other health professionals:*

*‘Knowing what your role is and sticking to that, I suppose being assertive with other disciplines.’* [47]

### Theme 2b; training

Training was seen as an important tool that could increase practitioner resilience. However, there was a sense that training was often inadequate for these complex roles (CMOC 13). Nevertheless, there was evidence to support, or recommend, training. Training could be aimed at developing resilience, training for the roles more generally and training directed at the complexity of the primary care patient presentation. With specific reference to the FCPP role, training to manage diagnostic/clinical uncertainty was suggested (CMOC 14–16).

### Theme 2c; experience

Experience, ‘clinical mileage’ or years in practice were often described as mitigating burnout. It was felt that practitioners with limited experience would be at greater risk of burnout due to the stress, and worry, they would be likely to experience these complex roles (CMOC 18, 19).

*Anxiety due to uncertainty and fear of bad outcome is most apparent in younger and less experienced staff* [20]

*[B]urnout was perceived as a risk factor for first contact physiotherapists possessing limited advanced practice experience’* [21]

Beyond more general ‘years of practice’ there was evidence to suggest greater resilience developed with prior experience of other similarly complex roles (CMOC 17, 20). For physiotherapists working in FCPP roles it also appeared that a broad early clinical exposure and advanced practice experience increased their confidence and better equipped them for coping in these complex primary care roles (CMOC 17, 20).

*Other aspects of relevant post-registration work experience were rotating PT posts across different clinical areas (e.g. cardiorespiratory and neurology)…“For me, it was when I was working in the rotations and doing the core areas of physio. The knowledge of all those core areas, means you are not fazed when a patient presents with comorbidities”.* [49]

## Theme 3; Complexity

It was clear that an inherent characteristic of these novel primary care-based roles was caseload complexity [14, 15, 18, 21, 45, 50]. ‘Participants highlighted that in their FCP role, they were dealing with more complex patients, which they had not anticipated at the outset’ [14].

*I am seeing stuff that would have gone straight to a rheumatology clinic; but now you’re the first person that is picking that up and doing the primary diagnosis. I’ve had a first diagnosis of multiple sclerosis in clinic; so quite a wide range of pathologies, but you need to have the clinical skills to recognise and know how to appropriately onward manage them… it’s the level of clinical complexity that you’re dealing with.* [14]

The lack of clear role boundaries and role definitions led to inappropriate referrals, greater caseload, clinical complexity with patients often presenting with complex multimorbidity and medical conditions outside the scope of the practitioner [14, 18, 21, 45, 51].

### Theme 3a; Caseload complexity, burden of responsibility, stress (CMOC 21–23)

The caseload encountered in general practice was, by its nature, complex. Psychosocial and socioeconomic characteristics, added to this complexity, as did the lack of clearly defined role boundaries and definitions. These challenged practitioners’ confidence because they were anxious about their competence to cope with this complexity and were worried about making a mistake (CMOC 22, 23). For physiotherapists working in FCPP roles the innate uncertainty of the caseload added to their worry, and, for some, this could lead to them having turnover intentions (CMOC 23).

*The worry connected with uncertainty seemed too much for some who had decided to leave their role altogether: I think I have made the decision that I am going to look for something else, because you can't be going home every day worrying about patients and worrying about your job, you are going to end up having a breakdown. (FCP 7) *[18]

### Theme 3b; Nature of role, caseload complexity, practical and emotional consequences (CMOC 24, 25)

It appeared that a fundamental aspect of these novel primary care roles was the reduced time that practitioners experienced compared to their previous roles.

*I was speaking to FCPs in [name of English region] and Wales, they were on 20 min[utes] and they were always overrunning by half an hour, or an hour, every day. Is that good for you to be doing that every day? I know the GPs do that and that is one of the main reasons we’re there, because they’re struggling to recruit GPs and, because of burnout, they’re going part-time. So, if we wanted it to be a full-time FCP, we’ve got to look after our health and wellbeing as well. P10. *[14]

While this was justified based on efficiency the consequences included clinics overrunning and practitioners experiencing greater levels of stress (CMOC 24). Paradoxically, the steps practitioners took to manage their caseload complexity (for example, ordering diagnostic tests, consulting with peers and GPs (*In those instances, further advice from a physician was needed to order appropriate investigations and complete paperwork, which frequently led to overrunning. *[21], utilising a biopsychosocial approach) often took longer and added to their burden both at a practical level and emotionally (CMOC 25).

## Theme 4; Working environment

The theme ‘Working Environment’ is made up of several sub-themes, practitioner presence, practitioners’ sense of isolation, challenges to working practice and supervisory support.

### Theme 4a, Practitioner presence

Across all the literature, for all professional (and non-professional) groups it was evident that being co-located had several benefits (CMOC26, 27, 33). Co-location provided practitioners with greater accessibility to practice staff (CMOC 27) and more chances to share understanding of one another’s roles and resulted in greater access to support and more effective multidisciplinary work (CMOC 26).

*The impact of effective interprofessional collaboration was discussed significantly … where daily first contact physiotherapy cover was provided [emphasis added]. Effective collaboration was thought to increase the knowledge base of the first contact physiotherapists, providing them with a greater awareness of medical conditions and the investigations required to diagnose them. This meant that healthcare efficiency was increased, and patients benefited *[21]

### Theme 4b, Practitioners’ sense of isolation

Although co-location facilitated integration it was apparent from the data that these novel, complex roles were often inherently isolating for practitioners. Often practitioners contrasted the roles from their previous roles and described the lack of team ethos and support (CMOC 28).

*You can feel quite isolated in your FCP role. Most of my career I’ve worked in a team and …we need support, emotional support. You know, it’s you in your four walls. The GPs they’re all very supportive, but they’re behind four walls as well. There’s very little interaction; there’s no meeting on a person-to-person level. Lunchtimes don’t overlap, so, you’re not getting to know these people. *[14]

The isolation and subsequent lack of belonging that practitioners experienced may lead them to considering leaving their role because the role was not what they were expecting it to be (CMOC 28, 29).

*I think moving from a well‐knit, very socially accepted and accessible department feeling within physiotherapy outpatients to then being kind of a bit more isolated and out of the way. That's some of the reasoning behind some of my colleagues sidestepping away from FCP. (FCP 5). *[18]

The data suggested a proportionality between co-location/physical presence and perceived effectiveness. Essentially, greater presence was correlated with greater effectiveness (CMOC 26, 27).

#### More presence

*Practices that fully integrated the LWP (Link Worker Programme) had a better shared understanding of the programme, higher staff engagement, and implemented the LWP at all three of its intended levels of impact (patient, practice, and community). *[52]

It was also evident from the data that modern working practices (online working) and wider operational issues (commissioning practices) challenged practitioner integration and undermined the perceived benefits of co-located working and risked further isolating practitioners (CMOC 29). These modern working practices extended to, and impacted, all general practice staff.

*GPs are spending more time in their rooms processing online consultations, increasing isolation and reducing informal interaction between staff and have larger workloads as a consequence of new administration tasks. *[53]

Several systems were described as having the potential to support practitioners and, consequently, reduce the sense of isolation (CMOC 30). These included practitioners working in portfolio roles (where some time was spent as an FCPP and some time working in general physiotherapy clinics) and practitioners working as part of a wider MSK team. Other mitigating features had arisen in response to other operational challenges such as the Covid-19 pandemic (virtual practice learning communities, training programmes and remote learning systems).

*Utilising Project ECHO for FCP CPD has the potential to reduce the isolation of clinicians working in Primary Care by creating a virtual community of practice, promoting knowledge exchange and improving clinician job satisfaction and patient care *[54]

These systems were described as allowing practitioners to feel more supported and improved resilience (CMOC 30).

### Theme 4c, Challenges to working practice

Alternatively, it appeared that there were several ways in which services were set up that added to practitioner burden. Resultingly, practitioners were left feeling professionally compromised and that they were being asked to do what wasn’t feasible (CMOC 31, 32). There were multiple examples that included:

#### Service capacity

Service capacity was less than demand resulting in sub-optimal impacts demonstrated by services and the development of waiting lists.

*So, I’ve got a 6-week waiting list, which as a FCP, shouldn’t exist, I’m not here in a physio role. And I don’t think they get that. P2 *[14]

#### Appointment length

Shortened appointment times not allowing sufficient time for the complexity of the practitioners’ caseload. This was described as detrimentally impacting patient care, increasing risk and compromising practitioner resilience/mental health.

*If you're time constrained, and your behind and you've got letters and scans and bits and pieces to do actually are you given that next patient your full attention? Or are you so wrapped up in all the other pressures and things that you've got to do that sort of drop the ball for a second. (FCP 3). *[18]

#### Administrative factors

Several compromising administrative factors were described, which increased practitioner burden and could ultimately lead to practitioner burnout and high staff turnover. A direct correlation between lack of administration time and burnout was described [22]. Beyond that straightforward relationship, governance and infrastructure shortcomings were seen to inhibit practitioners.

*NHS governance was also highlighted as impacting their role: For example, still to this day, I can’t order an x-ray. And we’re getting to the point now, where I’m feeling it may affect patient safety… It’s like boxing with no hands. We haven’t got the tools to do the job. *[14]

#### System-wide factors

Broader, system-wide factors were also described as impacting practitioners’ work experience. These factors ranged from a lack of understanding of services resulting in services being misused, through to funding concerns. Funding concerns related to lack of ring-fenced funding to support GP mentorship/supervision, lack of funding limiting service capacity, and lack of wider system funding resulting in a reduction in alternative services to support patients and practitioners’ onward referral options.

*The lived experience of link workers, as depicted in papers included in this review, highlights how the state of the wider health and care system both impacts their workloads and their role stress due to the fact that they may find themselves “holding” service users who they cannot connect onwards due to relevant services or support either not being present or having limited capacity. *[45]

### Theme 4 d, supervision

Despite the challenges described, at times, in securing supervision this was seen as one of the most important protective factors that supported practitioners. Supervision was described in several forms (online, via messaging applications, formal, informal) but supported practitioner clinical practice, mitigated uncertainty and supported practitioner well-being (CMOC 33). It was also evident that alongside the other benefits of being co-located, it also supported access to supervision (CMOC 33).

*Access to appropriate supervision and support networks across all employment models was seen as vital to aid both decision making and wellbeing *[18]

*Participants felt that co-location was an important facilitator in informal supervision, which would be less easily achieved through supervision carried out collaboratively with neighbouring practices. *[46]

## Discussion

To the authors knowledge this is the first realist review exploring the experiences of FCPPs with the explicit intention of providing recommendations about the support needs of these clinicians. When faced with the evidence of burnout in this population [18, 22, 55] and the objectives of the NHS Longterm workforce Plan [28] this becomes an imperative.

The findings of this realist review reveal a dynamic, multi-layered system in which the four themes synthesised (role characteristics, personal capacities, caseload complexity, and the working environment) interact to influence practitioner experiences. Of relevance to our primary objective, at the heart of these interrelations lies a central challenge: the consistent imbalance between professional expectations and the reality of the roles that they experience, which contributes to anxiety, stress, frustration, risking burnout and ultimately turnover intentions.

Role characteristics were typified by role ambiguity- where role definitions and role boundaries were unclear, in turn leading to misaligned expectations, role boundary drift, and clinical risk. This emerged as a foundational driver and it was impactful throughout the remaining themes. As demonstrated in CMOCs 1–8, unclear role definitions and role boundaries can lead to established practice staff misunderstanding a practitioner’s remit, resulting in patients that are inappropriate for first contact practitioners in practitioners’ caseloads and unrealistic expectations. This leads to additional caseload complexity (CMOCs 21–23), with practitioners expected, or required, to manage patients outside their competence or expected scope. These pressures, and the associated job discrepancy, manifest in anxiety, fear of clinical error, and erosion of professional identity- common predictors of burnout and turnover intention [56–59].

Resilience is defined as a mediator of burnout [60] with some individuals having inherent characteristics that support resilience. However, the broad nature of the role stressors means that once complexity increases, even the most experienced and qualified practitioners face limits to their tolerance. Exposure to manageable, challenging situations can build resilience and the confidence to articulate role boundaries (CMOCs 10–12), but resilience is limited. The evidence suggested that less experienced practitioners were more susceptible to worry, stress and burnout (CMOCs 18, 19). This links with previous research that correlates lack of experience to burnout [61, 62]. Without adequate support- be it through training (CMOCs 13–16) or supervision (CMOCs 32–33)- practitioners described feeling professionally compromised and emotionally depleted, echoing findings in Tierney et al. [59] and Leary et al. [63].

It was particularly apparent that working models amplified or somewhat mitigated these challenges. Non-co-located practitioners or practitioners with minimal presence in a practice were more likely to experience isolation (CMOCs 26- 29), which not only diminished team cohesion but also undermined access to peer support and informal supervision (CMOCs 26–27). This mirrors previous evidence [25, 64] that isolation and limited social capital were predictive of job dissatisfaction and burnout.

Indicative of the nature of themes, it was apparent that both role definitions and boundaries, and caseload complexity interacted with one another. These novel roles were inherently complex but unclear role definitions and role boundaries further compounded the caseload complexity. As demonstrated in themes 1 and 3 this resulted in a perceived sense of increased patient risk (CMOC 8, 21–23) which practitioners were left to ‘hold’. It is easy to envisage this sense of risk, distress and uncertainty being further amplified with inadequate support and supervision (Theme 4, CMOCs 28–29, 31, 32) or when practitioners are less experienced (Theme 2, CMOC 18), and how this may lead to practitioner burnout and turnover (CMOCs 19, 23, 29). The paradox here is that mitigation strategies- spending more time per patient, making referrals, consulting supervisors- often exacerbated the burden, increasing both time pressure and emotional fatigue. This negative feedback loop aligns with the Job Demands–Resources (JD-R) model [65] and is corroborated by Cantu et al. [27] who found similar trends among a broad population of physical therapists.

As observed, all themes contributed to practitioner symptoms that are associated with burnout, whether this was clinical uncertainty and complexity causing stress, stress related to a lack of confidence, disillusionment because of role discrepancy [25, 58, 61]. Perhaps unsurprisingly, turnover intentions followed logically (CMOC 23). This aligns with previous research that correlates role discrepancy with both burnout and turnover intentions [56, 66, 67].

In sum, this review provides a systems-level understanding of how burnout, and subsequent turnover intentions, in novel primary care roles are not caused by isolated failings but by interconnected contextual, organizational and operational blind spots. Where organisational structures fail to provide clear roles, effective supervision, and manageable workloads, even highly resilient and experienced practitioners become vulnerable to attrition.

Addressing these issues requires integrated solutions: workforce planning that aligns caseload with capacity, interprofessional education to clarify boundaries, and investment in supervision and emotional support infrastructure. Only with such multi-level interventions can primary care roles be sustained in a way that safeguards both practitioner wellbeing and patient care.

Implications for policy and practice.

### Role characteristics

Sustainable implementation of these roles requires greater clarity around responsibilities, improved triage and signposting processes, and interprofessional education to align expectations across primary care teams.

### Personal characteristics

Practitioner resilience, which is an antidote to burnout, must be understood as a dynamic capability shaped by both individual traits and contextual factors, including structured training, experiential learning opportunities, and systemic support, which together enable practitioners to thrive and remain in these challenging roles.

### Complexity

Without clearer role definitions and role boundaries, adequate support, and structural changes to address time constraints and team understanding, the complexity inherent within these roles is amplified and this could threaten practitioner wellbeing and the sustainability of such workforce innovations.

### Work environment

Working environments that prioritise integration, supervision, and realistic operational support are essential to ensuring practitioner wellbeing, role effectiveness, and long-term retention.

### Strengths and limitations

A particular strength of this realist review is its novelty in exploring the relationship between the FCPP role and turnover intentions. This is a timely and important first step that acts as a stepping off point for a subsequent realist evaluation. A further strength is the inclusion of data from other professions who are enacting similar roles to that of FCPPs. The corroborative data, across professional groups, strengthens both the validity of the findings and their transferability. As such, this realist review could be of use to other workforce groups. The skills and experience of the project team was a strength as was the inclusion of a patient stakeholder group. However, it is acknowledged that there are methodological limitations in this realist review. The limited timeframe and human resource of the project meant that the search criteria had to be limited, and some sources may have been missed. It was also not possible to repeat the search, and this may have resulted in new data being missing. We identified two new publications [55, 59] which would have contributed data, if included. Reassuringly, these recent publications reinforce the findings of this review. A greater search of grey literature, including social media, may have enhanced the data. The searches were restricted to UK literature so the findings may not be transferable.

This review has explored the challenges faced by FCPPs with a specific lens on turnover intentions as a consequence thereof. This is a novel area of research with respect to FCPPs and warrants further investigation. The team are in the process of doing this through a mixed methods realist evaluation.

## Conclusion

This realist review highlights the multifaceted challenges faced by FCPPs working in primary care and the systemic factors contributing to burnout and turnover intentions. By synthesising 33 CMOCs across four interacting themes- role characteristics, personal capacities, caseload complexity, and working environment- this study reveals the cascading, cyclical relationships that undermine practitioner well-being and service sustainability.

This review underscores that burnout and turnover intentions in FCPPs are not isolated outcomes but emergent properties of complex, interacting contextual mechanisms. Solutions must therefore be equally systemic multi-level and include all stakeholder groups: clarifying roles and boundaries, strengthening team integration, providing robust supervision, and ensuring caseloads are matched to capacity.

## Supplementary Information

Supplementary Material 1.

Supplementary Material 2.

Supplementary Material 3.

Supplementary Material 4.

Supplementary Material 5.

Supplementary Material 6.

## Data Availability

The authors confirm that all data generated, synthesised and analysed during this review are included in this published article. Furthermore, sources and data supporting the findings of this study were all publicly available at the time of submission. Any further enquiries regarding data generated or analysed during this review are available on request.

## Declarations

The views expressed are those of the author(s) and not necessarily those of the NIHR or the Department of Health and Social Care.
