# Supplementary material for: Understanding the retention and support needs of UK first contact practitioner physiotherapists in primary care; a realist review
Source: BMC Prim Care. 2026 Feb 13;27:68. doi: 10.1186/s12875-026-03197-6 (PMC12918251; doi:10.1186/s12875-026-03197-6)
Supplement: Supplementary file 1 — Supplementary Material 1. [file 12875_2026_3197_MOESM1_ESM.docx]

**Additional File 1**

**Understanding the retention and support needs of UK first contact practitioner physiotherapists in primary care; a realist review**

**Example of search Strategy**

Search strategy, example as applied to Ovid, MEDLINE

Ovid MEDLINE(R) ALL <1946 to September 17, 2025>

| **Search terms** | **Number of search hits** |
| --- | --- |
| Physical Therapists/ | 4,140 |
| (physioth* or "physical therapist*").mp. | 51,060 |
| (FCPP or "first contact practitioner physiotherap*").mp. | 22 |
| exp Pharmacists/ | 24,243 |
| ("clinical pharmacist*" or "senior pharmacist*" or "pharmac* technician*" or pharmacist*).mp. | 55,194 |
| ("social prescrib*" or "social prescribing link worker*" or "link worker*").mp. | 733 |
| "care co?ordinator*".mp. | 1,148 |
| ("health coach*" or "well?being coach*").mp. | 1,802 |
| exp Occupational Therapists/ | 983 |
| "occupational therapist*".mp. | 8,544 |
| exp Paramedics/ | 311 |
| "paramedic*".mp. | 11,437 |
| podiatrist*.mp. | 1,201 |
| Nutritionists/ | 2,114 |
| dietitian*.mp. | 9,431 |
| Education, Nursing, Associate/ | 1,785 |
| "nursing associate*".mp. | 1,954 |
| "mental health practitioner*".mp. | 1,160 |
| Physicians, Primary Care/ | 4,854 |
| "physician* associate*".mp. | 697 |
| "general practice assistant*".mp. | 8 |
| "digital and transformation lead*".mp. | 5 |
| "advanced practitioner*".mp. | 746 |
| exp Allied Health Personnel/ | 56,765 |
| "allied health professional*".mp. | 3,176 |
| 1 or 2 or 3 or 4 or 5 or 6 or 7 or 8 or 9 or 10 or 11 or 12 or 13 or 14 or 15 or 16 or 17 or 18 or 19 or 20 or 21 or 22 or 23 or 24 or 25 | 198,874 |
| exp General Practice/ | 80,592 |
| "general practice*".mp. | 61,174 |
| exp Primary Health Care/ | 208,615 |
| ("primary care" or "primary health care" or "primary healthcare").mp. | 225,574 |
| Patient-Centered Care/ | 25,209 |
| ("person-centred care" or "patient-centred care").mp. | 7,502 |
| exp Family Practice/ | 67,999 |
| "family practice*".mp. | 72,089 |
| 27 or 28 or 29 or 30 or 31 or 32 or 33 or 34 | 420,385 |
| exp Burnout, Psychological/ | 21,037 |
| Stress, Physiological/ | 91,146 |
| ((psychological or profession* or work or practitioner*) adj7 (burnout or "burning out" or fatigu* or pressure* or uncertaint* or stress* or overwhelm*)).mp. | 218,782 |
| ((staff or workforce or employe* or personnel or role*) adj5 (retention or turnover or resign* or quit* or leav*)).mp. | 19,370 |
| ((job or workplace or work or employee or employment or career) adj7 (well?being or satisf* or enjoy* or engag* or content*)).mp. | 62,136 |
| exp Anxiety/ | 129,770 |
| 36 or 37 or 38 or 39 or 40 or 41 | 477,684 |
| 26 and 35 and 42 | 885 |
| limit 43 to yr="2000 -Current" | 764 |
