# Supplementary material for: Understanding the retention and support needs of UK first contact practitioner physiotherapists in primary care; a realist review
Source: BMC Prim Care. 2026 Feb 13;27:68. doi: 10.1186/s12875-026-03197-6 (PMC12918251; doi:10.1186/s12875-026-03197-6)
Supplement: Supplementary file 2 — Supplementary Material 2. [file 12875_2026_3197_MOESM2_ESM.docx]

**Additional File 2**

**Table of included studies**

| Author/Year | Title | Role focus | Design | Sample |
| --- | --- | --- | --- | --- |
| Alghamdi et al., 2020 | Independent pharmacist prescribers views of their role as prescribers in primary care settings in Wales | Pharmacists | Conference abstract (survey) | 10 independent pharmacist prescribers (Wales) |
| Alshehri et al., 2023 | Integration of pharmacist independent prescribers into general practice: a mixed-methods study of pharmacists’ and patients’ views | Pharmacists | Mixed methods (interviews + questionnaire) | 13 independent pharmacist prescribers, patients 77 |
| Anchors et al., 2023 | The impact of remote consultations on the health and wellbeing of first contact practitioners | FCPP | Mixed methods (survey + interviews) | 109 survey, 16 interviews |
| Ashton, 2020 | Does medical uncertainty affect physiotherapist practitioners working within a first contact role? | FCPP | Survey study | 15 physiotherapist FCPPs |
| Bartlett., 2022 | Practice-based pharmacists: considerations for general practices | Pharmacists | Discussion paper / considerations | NA |
| Bassett & Jackson, 2020 | Challenges and learning opportunities of pre-registration physiotherapy placements in FCPP settings | FCPP | Qualitative interviews | 15 MSK FCPPs |
| Bassett & Jackson, 2022 | The professional development and career journey into musculoskeletal first contact physiotherapy | FCPP | Qualitative telephone interviews | 15 MSK FCPPs |
| Bicker et al., 2024 | A narrative synthesis of the effectiveness and acceptability of musculoskeletal FCPP roles in primary care | FCPP | Narrative synthesis (review) | 8 studies |
| Bowden et al., 2014 | Working on the Edge: Stresses and Rewards of Work in a Front-line Mental Health Service | Link workers | Qualitative study (interviews, focus groups) | 9 Front-line mental health link workers |
| Campbell et al., 2024 | Using Twitter (X) to mobilize knowledge for First Contact Physiotherapists: a qualitative study | FCPP | Qualitative interviews | 19 FCPPs (England) |
| Chng et al., 2021 | Implementing social prescribing in primary care in deprived areas (Deep End Links Worker Programme) | Link workers | Process evaluation (qualitative case study) | Lead GPs, link workers   - Focus groups 31 participants - Interviews with lead staff 14 participants - End-of-evaluation interview with lead staff 19 participants |
| Empson & May, 2024 | The perceptions of first point of contact roles in primary care: pre-registration students of the allied health professions | FCPP | Qualitative focus groups | 22 AHP students |
| Francetic et al., 2022 | Skill-mix change and outcomes in primary care: Longitudinal analysis of general practices in England 2015-2019 | Primary care workforce | Longitudinal analysis | NA |
| Frost et al., 2022 | Advanced Practice Physiotherapists and the implementation of the JIGSAW-E model for osteoarthritis management in Scottish primary care | APP / FCPP | Qualitative interviews + workshop | 6 general practitioners  8 APPs were interviewed.  23 practitioners attended the workshop |
| Goodwin et al., 2021 | Evaluation of the First Contact Physiotherapy (FCPP) model of primary care: qualitative insight | FCPP | Qualitative evaluation | 39 participants (patients, GPs, FCPPs, staff) |
| Goodwin et al., 2023 | Dealing with uncertainty as a first contact practitioner: a mixed-methods evaluation | FCPP | Mixed methods (survey + focus groups) | FCPPs  19 survey response  Two focus groups of 8 FCPPs |
| Greenhalgh et al., 2020 | A qualitative study to explore the experiences of first contact physiotherapy practitioners in the NHS and their experiences of their role | FCPP | Qualitative study | 10 FCPPs (Northwest England) |
| Griffith et al., 2021 | Link working at the intersections: an ethnographic exploration of delivering social prescribing in primary care | Link workers | Ethnographic study | Link workers  24 observations,  5 interviews,  Three focus groups with 17 participants |
| Halls et al., 2020 | Provision of first contact physiotherapy in primary care across the UK: a survey of the service | FCPP | National survey | 102 survey responses  32 service managers  70 FCPPs |
| Hassan et al., 2023 | An exploration of general practice pharmacists views on their role in general practice: a cross-sectional questionnaire study | Pharmacists | Cross-sectional questionnaire | 155 GPPs |
| Ingram et al., 2021 | Implementation of a provider-based musculoskeletal first contact physiotherapy service | FCPP | Implementation case study | NA |
| Ingram et al., 2023 | The experiences of uncertainty amongst musculoskeletal physiotherapists in first contact practitioner roles | FCPP | Qualitative phenomenology | 8 MSK FCPPs |
| Ingram et al., 2024 | The experiences of uncertainty amongst Musculoskeletal Physiotherapists in First Contact Practitioner roles (conference abstract) | FCPP | Qualitative phenomenology (conference abstract) | 8 MSK FCPPs |
| Iqbal & Allinson, 2019 | Evaluation of pharmacists working in a GP practice in South Wales | Pharmacists | Qualitative interviews | 2 pharmacists, 7 GPs |
| Lamb et al., 2023 | Patient access to first contact practitioner physiotherapists in the UK: a national survey | FCPP | Survey study | 193 patients |
| Lamb et al., 2024 | The experiences of patients with musculoskeletal conditions accessing first contact physiotherapy in the UK | FCPP | Qualitative interviews | 13 patients |
| Langridge, 2019 | The skills, knowledge and attributes needed as a first contact physiotherapist in primary care | FCPP | Qualitative study | 11 MSK FCPP physiotherapists |
| Lewis & Gill, 2023 | Facilitators and barriers regarding the implementation and interprofessional collaboration of a first contact physiotherapy service in Wales | FCPP | Qualitative interviews | 11 FCPPs (Wales) |
| Manson et al., 2021 | Enhancing first contact practitioners CPD within an integrated care system using Project ECHO | FCPP | CPD programme evaluation (survey) | 41 FCPPs who attended Project ECHO |
| Maskrey et al., 2022 | Releasing GP capacity with pharmacy prescribing support and new ways of working: a prospective observational cohort study | Pharmacists | Prospective observational cohort | General practice staff  Baseline- 36 participants  Follow-up- 63 participants |
| Millington et al., 2024 | Factors that influence the quality of the clinical supervision experience in an FCPP role | FCPP | Qualitative study (interviews) | 12 semi-structured group interviews of supervisors and supervisees in FCPP roles |
| Mohamed & Alldred, 2023 | A service evaluation and stakeholder perspectives of the Pharmacy Support and Development Service across a GP Federation | Pharmacists | Service evaluation (survey) | 8 GP managers, 26 pharmacy staff |
| Morcos & Dalton, 2021 | Exploring pharmacists perceptions of integrating pharmacists into the general practice setting (Ireland) | Pharmacists | Qualitative interviews | 12 community pharmacists (Ireland) |
| Morris et al., 2020 | Patient acceptability of the physiotherapy first contact practitioner role in primary care | FCPP | Realist evaluation (qualitative interviews) | 20 patients |
| Mueller et al., 2021 | Competencies required for general practice clinical pharmacists providing the Scottish pharmacotherapy service | Pharmacists | Mixed methods competency study | 20 Scottish general practice pharmacists |
| Muldoon and Seenan., 2023 | The introduction of advanced paramedics into primary care in Northern Ireland: a qualitative descriptive study of the experiences of GPs | Advanced paramedics | Qualitative descriptive study | 4 General practitioners (Northern Ireland) |
| Nabhani-Gebara et al., 2020 | General practice pharmacists in England: Integration, mediation and professional dynamics | Pharmacists | Case study (qualitative) | 10 GP surgeries |
| Nelson et al., 2022 | Scale, scope and impact of skill mix change in primary care in England: a mixed-methods study | Primary care workforce | Mixed methods | General practices in England  Pharmacists 19  GPs 9  Nurses 9 |
| Noseda, 2023 | What is the prevalence of burnout in UK primary care? | FCPP | Survey (systematic review of burnout prevalence) | 332 survey responses |
| Ryan et al., 2023 | Pharmacists in general practice: a qualitative interview case study of stakeholders experiences in a West London GP federation | Pharmacists | Qualitative interviews (case study) | 47 Stakeholders in GP federation |
| Turk et al., 2024 | A meta-ethnography of the factors that shape link workers experiences of social prescribing | Link workers | Meta-ethnography (systematic qualitative synthesis) | 21 qualitative studies |
| Matheson et al., 2022 | Resilience of primary healthcare professionals working in challenging environments: a focus group study | Primary care workforce | Focus groups | Five focus groups 20 health  Professionals  6 GPs  9 nurses  4 pharmacists  1 practice manager |
| Williams, 2023 | The value of virtual professional learning communities to support clinical education and telehealth practice | FCPP | Qualitative case study (virtual PLC) | NA |
