# Supplementary material for: Understanding the retention and support needs of UK first contact practitioner physiotherapists in primary care; a realist review
Source: BMC Prim Care. 2026 Feb 13;27:68. doi: 10.1186/s12875-026-03197-6 (PMC12918251; doi:10.1186/s12875-026-03197-6)
Supplement: Supplementary file 3 — Supplementary Material 3. [file 12875_2026_3197_MOESM3_ESM.docx]

Additional File 3

PRISMA Diagram; Support Needs of First Contact Practitioner Physiotherapists

Studies from databases/registers **(n = 3180)**

Embase (n = 1150)

PsycINFO (n = 817)

MEDLINE (n = 764)

CINAHL (n = 449)

**Identification**

References removed **(n = 409)**

Duplicates identified manually (n = 1)

Duplicates identified by Covidence (n = 408)

Marked as ineligible by automation tools (n = 0)

Studies excluded **(n = 33)**

Not UK/ NHS (n = 5)

Not primary care (n = 4)

physician/ GP/ Nurse (n = 13)

abstract- insufficient detail (n = 2)

Not relevant to programme theories (n = 7)

No relevance to first contact intervention (n = 1)

Community not primary care/ general practice (n = 1)

Studies screened **(n = 2771)**

Studies sought for retrieval **(n = 76)**

Studies not retrieved **(n = 0)**

Studies excluded **(n = 2695)**

Studies assessed for eligibility **(n = 76)**

**Screening**

Studies included in review **(n = 43)**

**Included**
