# Supplementary material for: Understanding the retention and support needs of UK first contact practitioner physiotherapists in primary care; a realist review
Source: BMC Prim Care. 2026 Feb 13;27:68. doi: 10.1186/s12875-026-03197-6 (PMC12918251; doi:10.1186/s12875-026-03197-6)
Supplement: Supplementary file 4 — Supplementary Material 4. [file 12875_2026_3197_MOESM4_ESM.docx]

**Additional File 4**

**Full list of refined CMOCs**

**Understand the retention and support needs of first contact practitioner physiotherapists in primary care- a realist review**

**Theme 1; Role Characteristics**

Role boundaries, confusion, inappropriate referrals (CMOCs 1-5)

1. When practitioners act as a first point of contact in their role in primary care (C) they will inevitably have to see patients that might find challenging to manage (O) because of the nature of untriaged patients (M).
2. When role definitions and role boundaries are unclear to practice staff (C) it leads to patients who are not appropriate for first contact practitioners in practitioners’ caseloads (O) due to practice staff uncertainty (M)
3. When role definitions and role boundaries are unclear to practice staff (C) it leads to patients who are not appropriate for first contact practitioners in practitioners’ caseloads (O) due to unrealistic expectations of other practice staff (M)
4. When Practitioners are being asked to see patients which they judge to be inappropriate for their first contact role (C), they are likely to become frustrated (O) because this is not what they expect to be doing (M)
5. When unclear role boundaries and role definitions result in patients in practitioners’ caseloads which have greater complexity (C) this may lead to practitioner stress (O) because they lack confidence in managing such patients (M)

Boundaries, referrer confusion, overwhelm/ overload (CMOC 6)

1. The increase in volume of patients and the increase in caseload complexity that arises when role boundaries and role definitions are unclear (C) may risk overwhelming services (O) because the time needed to deal with these exceed capacity (M)

Boundaries, scope of practice (CMOCs 7-8)

1. When Practitioners are faced with managing patients outside of their scope of their professional competence (C) they may still decide to practice outside the scope of their professional competence (O) because they feel under a professional obligation to do so (M)
2. When Practitioners are faced with managing patients outside of their scope of professional competence (C) there is a potential increase in risk to patients (O) because practitioners are less likely to have the knowledge and skills needed to manage such patients (M)

Role overlap, rivalries, tensions (CMOC 9)

1. Unclear role boundaries and role definitions, leading to role overlap between professions (C) may lead to interprofessional tensions (O) because of professional rivalries (‘turf wars’) (M).

**Theme 2; Personal characteristics**

Exposure to stress, opportunities to learn, resilience (CMOC 10)

1. When practitioners have been exposed to, and successfully managed stressful situations / circumstances (C), they develop resilience (O) because these situations have provided them with opportunities to learn from (M)

Resilience, confidence, boundary articulation (CMOCs 11-12)

1. Practitioners who have greater experience of exposure to manageable stress and who possess greater resilience (C) have learnt where their boundaries are (M) allowing them to articulate their boundaries (O)
2. When practitioners have had more exposure to and successfully managed more challenging patients (C) they are better able to understand where the boundaries of their practice are (O) because they have been able to learn from these experiences (M)

Training (CMOCs 13-15)

1. When adequate training, required for the complexity of novel primary care roles is not provided (C) practitioners experience high levels of stress (O) because they do not feel prepared for the roles (M)
2. When practitioners new to primary care have been provided with adequate training to develop appropriate skills needed to manage challenges in their new role (C) they are more likely to cope better with the challenges they face (O) because they have the confidence to do so (M)
3. If practitioners working as first contact practitioners have suitable training about how to deal with the diagnostic/ medical uncertainty related to the first contact consultation in general practice (C) they are more likely to cope better with the undifferentiated nature of the patient presentation (O) because they are more comfortable with uncertainty (M)

Experience, confidence, coping

Lack of experience, worry, stress (CMOCs 16-18)

1. When practitioners can draw on their previous experience, that is relevant to the challenges of complex primary care roles (C), they are more likely to cope better in the role (O) because they are more comfortable in their personal capabilities (M)
2. When practitioners have limited relevant experience of what is required within these complex primary care roles (C) they will experience more stress and anxiety (O) because they are worried about doing the wrong thing (M)
3. When practitioners are worried about doing the wrong thing due to limited relevant experience (C) they are greater risk of burnout (O) due to their feelings of stress/ anxiety (M)

Experience of exposure to conditions outside immediate professional scope of practice that are encountered in primary care roles, confident, coping

Experience of working at a professional advanced level of practice, confident, coping (CMOC 19)

1. Where practitioners have had experience of exposure to conditions encountered within primary care roles that are outside their immediate professional scope of practice (C) they feel more able to cope (O) because they are more confident about what to do (M)

**Theme 3; Complexity**

Caseload complexity, burden of responsibility, stress (CMOCs 20-22)

1. When practitioners have to manage patients with conditions that they are less familiar with managing (C) this can cause them stress (O) because they are not confident that they can manage these patients appropriately (M).
2. When practitioners’ role boundaries and role definitions are unclear it adds to the conditions that practitioners are less familiar with managing, or they feel are outside their scope of practice (C) and this can cause them stress (O) because they are not confident that they can manage these patients appropriately (M).
3. The diagnostic uncertainty associated with the undifferentiated patient that forms a fundamental part of the first contact role (C) causes practitioners to have turnover intentions (O) because they are worried about the consequences of making a mistake (M)

Nature of role, caseload complexity, practical and emotional consequences (CMOCs 23-24)

1. When practitioners are not provided with the time they judge they need to appropriately manage a patient with complex problems (C), they may become distressed (O) because they feel they are not doing their job properly (M)
2. When practitioners have to do more work to manage the caseload complexity and reduce diagnostic uncertainty that they experience in their role (C), these approaches usually result in clinics over-running (O) because these approaches often take up more time (M)

**Theme 4; Working Environment**

Presence (CMOCs 25-26)

1. When existing practice staff get more opportunities to interact with the ‘new’ practitioner (e.g. because they are visible and co-located in a practice) (C) this leads to more effective multiprofessional working (O) because staff get more chances to understand what each other can do (M)
2. When practitioners are co-located in their new role (C), they find it easier to get and give support and help when they need it (O), because people are more physically accessible (corridor chats, staff meetings, MDT meetings, lunchtime) (M)

Isolation (CMOCs 27-30)

1. If practitioners don’t experience a team ethos that supports them in their new clinical setting (C) they feel isolated/ scared (O) because they don’t feel they belong (M)
2. The isolation that practitioners experience when working in these novel primary care roles (C) may lead practitioners to consider leaving the role (O) because the role is not what they expected it to be (M)
3. When there are systems in place to help practitioners overcome the challenges of isolating work practices (online consultations/ hub model) (C) it aids the development of practitioner resilience (O) because they feel more able to cope (M)
4. When practitioners are made to work in ways that they believe are clinically inappropriate (C) they get frustrated (O) because they feel this is professionally compromising (M)

Capacity (CMOC 31)

1. When services are set up in ways that make it challenging for the practitioner to carry out what they think is expected of them (C), they get frustrated (O) because they feel they are being asked to do the impossible (M)

Supervision (CMOCs 32-33)

1. When suitable supervision is provided to practitioners in ways that they can access (C) they feel more able to undertake their role (O) because they feel supported (M)
2. Co-location provides greater potential access to both formal and informal supervision (C) which supports practitioners in their roles (O) as it provides them with the support, they feel they need (M)

Abbreviations

MDT, multidisciplinary team
