# Supplementary material for: Understanding the retention and support needs of UK first contact practitioner physiotherapists in primary care; a realist review
Source: BMC Prim Care. 2026 Feb 13;27:68. doi: 10.1186/s12875-026-03197-6 (PMC12918251; doi:10.1186/s12875-026-03197-6)
Supplement: Supplementary file 5 — Supplementary Material 5. [file 12875_2026_3197_MOESM5_ESM.docx]

Additional File 5

Refined CMOCs and Sources

| **CMOC** | **Description** | **Sources** |
| --- | --- | --- |
| **Role Characteristics** | | |
| **CMOC 1** | When practitioners act as a first point of contact in their role in primary care (C) they will inevitably have to see patients that might find challenging to manage (O) because of the nature of untriaged patients (M). | (1–4) |
| **CMOC 2** | When role definitions and role boundaries are unclear to practice staff (C) it leads to patients who are not appropriate for first contact practitioners in practitioners’ caseloads (O) due to practice staff uncertainty (M) | (1,2,5,6) |
| **CMOC 3** | When role definitions and role boundaries are unclear to practice staff (C) it leads to patients who are not appropriate for first contact practitioners in practitioners’ caseloads (O) due to unrealistic expectations of other practice staff (M) | (2,5,7,8) |
| **CMOC 4** | When Practitioners are being asked to see patients which they judge to be inappropriate for their first contact role (C), they are likely to become frustrated (O) because this is not what they expect to be doing (M) | (1,5,6) |
| **CMOC 5** | When unclear role boundaries and role definitions result in patients in practitioners’ caseloads which have greater complexity (C) this may lead to practitioner stress (O) because they lack confidence in managing such patients (M) | (1,5,7) |
| **CMOC 6** | The increase in volume of patients and the increase in caseload complexity that arises when role boundaries and role definitions are unclear (C) may risk overwhelming services (O) because the time needed to deal with these exceed capacity (M) | (1,2,6,9,10) |
| **CMOC 7** | When Practitioners are faced with managing patients outside of their scope of their professional competence (C) they may still decide to practice outside the scope of their professional competence (O) because they feel under a professional obligation to do so (M) | (3,5) |
| **CMOC 8** | When Practitioners are faced with managing patients outside of their scope of professional competence (C) there is a potential increase in risk to patients (O) because practitioners are less likely to have the knowledge and skills needed to manage such patients (M) | (3,7,11) |
| **CMOC 9** | Unclear role boundaries and role definitions, leading to role overlap between professions (C) may lead to interprofessional tensions (O) because of professional rivalries (‘turf wars’) (M). | (7,12–14) |
| **Personal Characteristics** | | |
| **CMOC 10** | When practitioners have been exposed to, and successfully managed stressful situations / circumstances (C), they develop resilience (O) because these situations have provided them with opportunities to learn from (M) | (1,15) |
| **CMOC 11** | Practitioners who have greater experience of exposure to manageable stress and who possess greater resilience (C) have learnt where their boundaries are (M) allowing them to articulate their boundaries (O) | (4,15) |
| **CMOC 12** | When practitioners have had more exposure to and successfully managed more challenging patients (C) they are better able to understand where the boundaries of their practice are (O) because they have been able to learn from these experiences (M) | (4,15) |
| **CMOC 13** | When adequate training, required for the complexity of novel primary care roles is not provided (C) practitioners experience high levels of stress (O) because they do not feel prepared for the roles (M) | (1,4,5,7) |
| **CMOC 14** | When practitioners, new to primary care have been provided with adequate training to develop appropriate skills needed to manage challenges in their new role (C) they are more likely to deliver safe and effective practice (O) because they have the confidence to do so (M) | (4,5,7,15–18) |
| **CMOC 15** | If practitioners working as first contact practitioners have suitable training about how to deal with the diagnostic/ medical uncertainty related to the first contact consultation in general practice (C) they are more likely to cope better with the undifferentiated nature of the patient presentation (O) because they are more comfortable with uncertainty (M) | (1,3,19) |
| **CMOC 16** | When practitioners can draw on their previous experience, that is relevant to the challenges of complex primary care roles (C), they are more likely to cope better in the role (O) because they are more comfortable in their personal capabilities (M) | (2,4,14,15) |
| **CMOC 17** | When practitioners have limited relevant experience of what is required within these complex primary care roles (C) they will experience more stress and anxiety (O) because they are worried about doing the wrong thing (M) | (2,4,14,15,19–21) |
| **CMOC 18** | When practitioners are worried about doing the wrong thing due to limited relevant experience (C) they are greater risk of burnout (O) due to their feelings of stress/ anxiety (M) | (1,20) |
| **CMOC 19** | Where practitioners have had experience of exposure to conditions encountered within primary care roles that are outside their immediate professional scope of practice (C) they feel more able to cope (O) because they are more confident about what to do (M). | (1–3,5,7,22,23) |
| **Complexity** | | |
| **CMOC 20** | When practitioners have to manage patients with conditions that they are less familiar with managing (C) this can cause them stress (O) because they are not confident that they can manage these patients appropriately (M). | (1,2,5,7,20,23) |
| **CMOC 21** | When practitioners’ role boundaries and role definitions are unclear it adds to the conditions that practitioners are less familiar with managing, or they feel are outside their scope of practice (C) and this can cause them stress (O) because they are not confident that they can manage these patients appropriately (M). | (1,2,7) |
| **CMOC 22** | The diagnostic uncertainty associated with the undifferentiated patient that forms a fundamental part of the first contact role (C) causes practitioners to have turnover intentions (O) because they are worried about the consequences of making a mistake (M) | (2,3,21,24) |
| **CMOC 23** | When practitioners are not provided with the time they judge they need to appropriately manage a patient with complex problems (C), they may become distressed (O) because they feel they are not doing their job properly (M) | (1,2,4,5,21,24) |
| **CMOC 24** | When practitioners have to do more work to manage the caseload complexity and reduce diagnostic uncertainty that they experience in their role (C), these approaches usually result in clinics over-running (O) because these approaches often take up more time (M). | (1–5,25) |
| **Working Environment** | | |
| **CMOC 25** | When existing practice staff get more opportunities to interact with the ‘new’ practitioner (e.g. because they are visible and co-located in a practice) (C) this leads to more effective multiprofessional working (O) because staff get more chances to understand what each other can do (M) | (1,5,7,9,13,14,16,24,26–29) |
| **CMOC 26** | When practitioners are co-located in their new role (C), they find it easier to get and give support and help when they need it (O), because people are more physically accessible (corridor chats, staff meetings, MDT meetings, lunchtime) (M) | (1,5,7,9,13,14,24,26–29) |
| **CMOC 27** | If practitioners don’t experience a team ethos that supports them in their new clinical setting (C) they feel isolated/ scared (O) because they don’t feel they belong (M) | (1–3,30,31) |
| **CMOC 28** | The isolation that practitioners experience when working in these novel primary care roles (C) may lead practitioners to consider leaving the role (O) because the role is not what they expected it to be (M) | (2,3,23) |
| **CMOC 29** | When there are systems in place to help practitioners overcome the challenges of isolating work practices (online consultations/ hub model) (C) it aids the development of practitioner resilience (O) because they feel more able to cope (M) | (9,30–34) |
| **CMOC 30** | When practitioners are made to work in ways that they believe are clinically inappropriate (C) they get frustrated (O) because they feel this is professionally compromising (M) | (13,20) |
| **CMOC 31** | When services are set up in ways that make it challenging for the practitioner to carry out what they think is expected of them (C), they get frustrated (O) because they feel they are being asked to do the impossible (M) | (1–3,5,6,14–16,21,23,24,27,35–37) |
| **CMOC 32** | When suitable supervision is provided to practitioners in ways that they can access (C) they feel more able to undertake their role (O) because they feel supported (M) | (3,9,13,36,38) |
| **CMOC 33** | Co-location provides greater potential access to both formal and informal supervision (C) which supports practitioners in their roles (O) as it provides them with the support, they feel they need (M) | (1,9,13) |

Abbreviations

MDT- multidisciplinary team

1. Lewis MW, Gill P. Facilitators and barriers regarding the implementation and interprofessional collaboration of a first contact physiotherapy service in primary care in Wales: A qualitative study. Int J Ther Rehabil. 2023 Jan 2;30(1).

2. Greenhalgh S, Selfe J, Yeowell G. A qualitative study to explore the experiences of first contact physiotherapy practitioners in the NHS and their experiences of their first contact role. Musculoskelet Sci Pract. 2020 Dec 1;50.

3. Ingram S, Stenner R, May S. The experiences of uncertainty amongst musculoskeletal physiotherapists in first contact practitioner roles within primary care. Musculoskeletal Care. 2023 Sep 1;21(3):644–54.

4. Langridge N. The skills, knowledge and attributes needed as a first-contact physiotherapist in musculoskeletal healthcare. Musculoskeletal Care. 2019 Jun 1;17(2):253–60.

5. Turk A, Tierney S, Hogan B, Mahtani KR, Pope C. A meta-ethnography of the factors that shape link workers’ experiences of social prescribing. BMC Med. 2024 Dec 1;22(1).

6. Lamb K, Comer C, Walsh N, McHugh G. Patient access to first contact practitioner physiotherapists in the UK: A national survey. Musculoskeletal Care. 2023 Dec 1;21(4):1554–62.

7. Alshehri AA, Hindi AMK, Cheema E, Sayeed Haque M, Jalal Z, Yahyouche A. Integration of pharmacist independent prescribers into general practice: a mixed-methods study of pharmacists’ and patients’ views. J Pharm Policy Pract. 2023 Dec 1;16(1).

8. Maskrey M, Johnson CF, Cormack J, Ryan M, Macdonald H. Releasing GP capacity with pharmacy prescribing support and New Ways of Working: A prospective observational cohort study. British Journal of General Practice. 2018 Oct 1;68(675):e735–42.

9. Bowden GE, Smith JCE, Parker PA, Boxall MJC. Working on the Edge: Stresses and Rewards of Work in a Front-line Mental Health Service. Clin Psychol Psychother. 2015 Nov 1;22(6):488–501.

10. Bartlett S. Practice-based pharmacists: considerations for general practices. Vol. 73, British Journal of General Practice. Royal College of General Practitioners; 2023. p. 249–50.

11. S. Alghamdi RD and KH. Independent pharmacist prescribers’ views of their role as prescribers in primary care settings in Wales. International Journal of Pharmacy Practice. 2020 Mar 31;28(Supplement_1):4–43.

12. Nabhani‐Gebara S, Fletcher S, Shamim A, May L, Butt N, Chagger S, et al. General practice pharmacists in England: Integration, mediation and professional dynamics. Research in Social and Administrative Pharmacy. 2020 Jan 1;16(1):17–24.

13. McDermott I, Spooner S, Goff M, Gibson J, Dalgarno E, Francetic I, et al. Scale, scope and impact of skill mix change in primary care in England: a mixed-methods study. Health and Social Care Delivery Research. 2022;10(9).

14. Morcos P, Dalton K. Exploring pharmacists’ perceptions of integrating pharmacists into the general practice setting. Exploratory Research in Clinical and Social Pharmacy. 2021 Jun 1;2.

15. Matheson C, Robertson HD, Elliott AM, Iversen L, Murchie P. Resilience of primary healthcare professionals working in challenging environments: A focus group study. British Journal of General Practice. 2016 Jul 1;66(648):e507–15.

16. A. Iqbal and M. Allinson. Evaluation of pharmacists working in a GP practice in South Wales. Vol. 63, British Journal of General Practice. 2013.

17. Mueller T, Preston KE, Mcfadyen Weir N, Bennie M, Newham R. Competencies required for General Practice Clinical Pharmacists providing the Scottish Pharmacotherapy Service: A modified eDelphi study. Health Soc Care Community. 2021 Nov 1;29(6):e328–37.

18. Muldoon D, Seenan C. The introduction of advanced paramedics into primary care in Northern Ireland: a qualitative descriptive study of the experiences of general practitioners. Br Paramed J. 2021 Dec 2;6(3):1–6.

19. Ashton L. Does medical uncertainty affect physiotherapist practitioners working within a ﬁrst contact role? An exploratory study. Physiotherapy. 2020 May;107:e158–9.

20. Anchors Z, Jones B, Thomas R, Berry A, Walsh N. The impact of remote consultations on the health and wellbeing of first contact physiotherapists in primary care: A mixed methods study. Musculoskeletal Care. 2023 Sep 1;21(3):655–66.

21. Bassett AM, Jackson J. Challenges and Learning Opportunities of Pre-Registration Physiotherapy Placements in First Contact Settings: The Perspectives of Musculoskeletal First Contact Physiotherapists. Musculoskeletal Care. 2020 Jun 1;18(2):140–9.

22. Bassett AM, Jackson J. The professional development and career journey into musculoskeletal first contact physiotherapy: a telephone interview study. Physiother Theory Pract. 2022;38(10):1453–68.

23. Bicker G, Hadley-Barrows T, Saunders A, Mairs H, Stevenson K. A narrative synthesis of the effectiveness and acceptability of musculoskeletal first contact physiotherapy practitioner roles in primary care. Vol. 22, Musculoskeletal Care. John Wiley and Sons Ltd; 2024.

24. Goodwin R, Moffatt F, Hendrick P, Stynes S, Bishop A, Logan P. Evaluation of the First Contact Physiotherapy (FCP) model of primary care: a qualitative insight. Physiotherapy (United Kingdom). 2021 Dec 1;113:209–16.

25. Goodwin R. HP, MF. Dealing with uncertainty as a first contact practitioner- a mixed methods evaluation. Physiotherapy. 2024 Jun;123:e114–5.

26. Ryan K, Patel N, Lau WM, Abu-Elmagd H, Stretch G, Pinney H. Pharmacists in general practice: A qualitative interview case study of stakeholders’ experiences in a West London GP federation. BMC Health Serv Res. 2018 Apr 2;18(1).

27. Morris L, Moule P, Pearson J, Foster D, Walsh N. Patient acceptability of the physiotherapy first contact practitioner role in primary care: A realist informed qualitative study. Musculoskeletal Care. 2021 Mar 1;19(1):38–51.

28. A. H. F. Hassan HEB and CMH. An exploration of general practice pharmacists’ (GPPs) views on their role in general practice a cross-sectional questionnaire study. 2023;

29. Chng NR, Hawkins K, Fitzpatrick B, O’Donnell CA, Mackenzie M, Wyke S, et al. Implementing social prescribing in primary care in areas of high socioeconomic deprivation: Process evaluation of the “Deep End” community Links Worker Programme. British Journal of General Practice. 2021 Dec 1;71(713):E912–20.

30. Manson J, Jackson H, Setchfield I. Enhancing first contact practitioners’ continuous professional development within an integrated care system using project ECHO (Extension for Community Health Outcomes). Physiotherapy. 2022 Feb;114:e89.

31. Campbell L, Quicke J, Stevenson K, Paskins Z, Dziedzic K, Swaithes L. Using Twitter (X) to Mobilize Knowledge for First Contact Physiotherapists: Qualitative Study. J Med Internet Res. 2024;26(1).

32. Ingram S, Stenner R, Acton T, Armitage K. Implementation of a provider based musculoskeletal first contact physiotherapy service model: Key points to consider. Musculoskeletal Care. 2021 Jun 1;19(2):232–5.

33. Mohamed H, Alldred S. A service evaluation and stakeholder perspectives of the “Pharmacy Support and Development” Service across a GP Federation [Internet]. Vol. 31, International Journal of Pharmacy Practice. 2023. Available from: https://www.

34. Williams R. THE VALUE OF VIRTUAL PROFESSIONAL LEARNING COMMUNITIES TO SUPPORT CLINICAL EDUCATION AND TELEHEALTH PRACTICE. Physiotherapy. 2024 Jun;123:e75–6.

35. Halls S, Thomas R, Stott H, Cupples ME, Kersten P, Cramp F, et al. Provision of first contact physiotherapy in primary care across the UK: a survey of the service. Physiotherapy (United Kingdom). 2020 Sep 1;108:2–9.

36. Nozedar L, O’Shea S. What is the prevalence of burnout amongst first contact physiotherapists working within primary care? Musculoskeletal Care. 2023 Sep 1;21(3):776–85.

37. Griffith B, Moffatt S, Pollard T. Link working at the intersections: an ethnographic exploration of delivering social prescribing in primary care. In: J Epidemiol Community Health. BMJ; 2021. p. A42.1-A42.

38. Francetic I, Gibson J, Spooner S, Checkland K, Sutton M. Skill-mix change and outcomes in primary care: Longitudinal analysis of general practices in England 2015–2019. Soc Sci Med. 2022 Sep 1;308.
