# Supplementary material for: Understanding the retention and support needs of UK first contact practitioner physiotherapists in primary care; a realist review
Source: BMC Prim Care. 2026 Feb 13;27:68. doi: 10.1186/s12875-026-03197-6 (PMC12918251; doi:10.1186/s12875-026-03197-6)
Supplement: Supplementary file 6 — Supplementary Material 6. [file 12875_2026_3197_MOESM6_ESM.docx]

**Additional File 6.**

**Refined CMOCs and supporting data, text extracts**

**Illustrative examples of evidence used to support CMOC development**

**Theme 1- Role Characteristics**

**Role boundaries, confusion, inappropriate referrals**

***Primary care role inherently complex***

*‘A total of five (45%) participants reported that they were often exposed to medical conditions, which required a broader knowledge base to identify that it was not a musculoskeletal issue. The impact of this exposure led some participants to lack confidence in managing patients independently, leading to heightened stress’. [1]*

*Participants highlighted that in their FCP role, they were dealing with more complex patients, which they had not anticipated at the outset: …* *‘It’s the level of clinical complexity that you’re dealing with’[2]*

*‘… taking on lots of new skills, lots of clinical encounters that are far more complex than you probably had to deal with as a band 6 clinician, you are no longer just [passing it on as it is complex], you’re dealing with it, to handle the clinical learning curve[1]*

*Uncertainty was prevalent in the role and seemed to be influenced by environmental factors, such as clinical complexity and time pressures [3]*

*Secondary care advanced MSK practice is historically linked to an orthopaedic pathway, and although safety is just as important here (FCPP), patients do not normally self‐refer to these services and so are commonly screened beforehand by a GP. For patients who are not initially screened, it is likely that the levels of uncertainty will be higher in many cases presented to a clinician. [4]*

***Unclear roles, confusion, unrealistic expectations, added complexity, stress/ emotional burden***

*However, there seemed to be some confusion about their role. This confusion was evident from patients, reception staff and GP*.[2]

*However, this same lack of clear role definition could cause role stress through workload overload and complexity. It could also lead to confusion/ambivalence among other members of staff about what social prescribing is and what the link worker role could offer, in turn contributing to inappropriate referrals*.[5]

*with “their job scope and remit being poorly defined from the outset” and their role being “not well understood by external referrers”. This could lead to unrealistic expectations or confusion around what is achievable through social prescribing and to the referral of patients with complex needs that exceeded the remit of the link worker role* [5]

*“However, these more involved approaches (complexity of patients and approaches used) may require additional time and training for link workers, and may cause high levels of stress and emotional burden* [5]

*“A lot of patients … will have polypharmacy, multimorbidity and this is where the individual Pharmacist has to make their own decision as to what they’re happy to prescribe or not prescribe … I think that can cause problems because if the GPs see the Pharmacist as an Independent Prescriber and … say the Hospital asks the GP surgery to prescribe a new medication. Usually, the GPs would expect that Pharmacist to prescribe that medication but then individually the pharmacist may feel that they’re not competent in that area.”* (PIP 5) [6]

‘*I feel GPs’ expectations of the team members I work with are very high. They expect them to take on too much for the limited time they have in practice.’ (New pharmacist, 10*) [7]

***Second contact consultation***

*A total of eight (73%) participants remarked that there was a lack of understanding of the first contact physiotherapist’s role, primarily among GPs, who perceived that first contact physiotherapists were offering secondary care physiotherapy services within primary care: ‘I feel that some GPs almost think of it [first contact physiotherapy] as there being a physiotherapy service within their surgery, rather than a screening or triage service.’ This caused duplication of care, with GPs providing musculoskeletal management in one appointment and then referring to the first contact physiotherapists for the same issue*. [1]

*A lack of role clarity existed, often leading to first contact physiotherapists acting as the second contact and the development of long waiting lists in some models, resulting in perceived inefficiencies that caused frustration*.[1]

*Duplication of appointments due to a lack of understanding about first contact roles was another common theme in the free text comments…There were several negative comments about FCPP appointments being used as ‘fast track’ or ‘in‐house physio’, often due to long waits for MSK outpatient services, which respondents felt were inappropriate or caused duplication of appointments[8]*

**CMOCs**

| **CMOC 1** | When practitioners act as a first point of contact in their role in primary care (C) they will inevitably have to see patients that might find challenging to manage (O) because of the nature of untriaged patients (M). |
| --- | --- |
| **CMOC 2** | When role definitions and role boundaries are unclear to practice staff (C) it leads to patients who are not appropriate for first contact practitioners in practitioners’ caseloads (O) due to practice staff uncertainty (M) |
| **CMOC 3** | When role definitions and role boundaries are unclear to practice staff (C) it leads to patients who are not appropriate for first contact practitioners in practitioners’ caseloads (O) due to unrealistic expectations of other practice staff (M) |
| **CMOC 4** | When Practitioners are being asked to see patients which they judge to be inappropriate for their first contact role (C), they are likely to become frustrated (O) because this is not what they expect to be doing (M) |
| **CMOC 5** | When unclear role boundaries and role definitions result in patients in practitioners’ caseloads which have greater complexity (C) this may lead to practitioner stress (O) because they lack confidence in managing such patients (M) |

**Boundaries, referrer confusion, overwhelm/ overload**

***Services overwhelmed***

‘*One of the issues encountered by the Linkworker Service was the lack of explicit and traditional boundaries for access developed for their new way of working and the linkworkers’ concern that they and their service would be overwhelmed’*.[9]

*Seven (64%) participants in models 1 and 3 stated that they were often reviewing patients as the second contact, which was not mentioned by participants in model 2. GPs often wishing to gain a second musculoskeletal opinion from the first contact physiotherapists contributed to the waiting lists in model 3*.[1]

*So, if anyone comes to see him [GP] with a back problem or a knee problem, he doesn’t deal with it, he sends them directly to me. So, I’ve got a 6-week waiting list, which as a FCP, shouldn’t exist, I’m not here in a physio role. And I don’t think they get that. P2*. [2]

*Some GPs certainly view us as fast access physio and misinform patient that they will have a course of physio*. [8]

*Their (pharmacists) expanding role and expertise present an ever-growing number of tasks that pharmacists can take on but if such workload is not appropriately managed and reviewed, they may quickly become overwhelmed and risk slipping into the realm of workload burden and burnout* [10]

**CMOC**

| **CMOC 6** | The increase in volume of patients and the increase in caseload complexity that arises when role boundaries and role definitions are unclear (C) may risk overwhelming services (O) because the time needed to deal with these exceed capacity (M) |
| --- | --- |

**Boundaries, scope of practice,**

***Scope of practice***

*‘Link workers were sometimes used to “fill gaps”, which is an identified risk of having unclear professional identities in newer professions. In the studies, parameters of the link worker role were frequently exceeded due to concerns that nobody would support patients otherwise, because “no other professions would deviate from their role boundaries*”[5]

*‘Some participants were keen to develop new skills and sought training opportunities on blood tests or non‐medical prescribing. The desire to improve knowledge and respond to service need, may inadvertently have contributed to the burden of responsibility and the poorly defined boundaries of the role: “Requesting or doing blood tests is a completely new thing… It just opened up pandora’s box honestly, because you say ‘okay now I realise how much I don't know”.’ (FCP 8*)[3]

*Some participants described situations, where PIPs had been asked to make decisions or carry out roles beyond their clinical competence due to GP practice teams’ lack of an understanding of their role. This was perceived to potentially jeopardise patient safety*.[6]

*Role clarity was also important to ensure that PIPs and their employing practice(s) are adequately covered by indemnity insurance, thereby providing confidence to the practice that they can safely and legally perform their responsibilities. “Another thing you have to think about all the time is like are you practicing with your scope? Is it within your competence? Is it safe what you are doing? You know, will your indemnity cover you for what you are doing?” (PIP 11).* [6]

*The lack of onward referral options or other appropriate sources of support may mean that link workers end up taking a more involved approach to patient care than originally anticipated. This may include taking on more risk than they have training for, due to having to deal with complex cases when there is no other support available* [5]

*some reported the topic of indemnity as a barrier, and they required more clarity in terms of what they are covered to prescribe and to do. [11]*

**CMOCs**

| **CMOC 7** | When Practitioners are faced with managing patients outside of their scope of their professional competence (C) they may still decide to practice outside the scope of their professional competence (O) because they feel under a professional obligation to do so (M) |
| --- | --- |
| **CMOC 8** | When Practitioners are faced with managing patients outside of their scope of professional competence (C) there is a potential increase in risk to patients (O) because practitioners are less likely to have the knowledge and skills needed to manage such patients (M) |

**Role overlap, rivalries, tensions**

***Professional rivalries and tensions***

*‘The data uncovered tension between medical staff and pharmacists, and the statements below reflect a lack of collaboration, and professional insecurity on behalf of both the doctors and pharmacists’* [12]

*In addition to this, was evidence of discord between pharmacists and nursing staff based around perceptions of role encroachment* [12]

*We have definitely found that with having the pharmacist that the overlap with the nursing role actually is quite dramatic you know in terms of doing asthma reviews, diabetic reviews, diabetes management. And so our pharmacist at the moment is …, well she has done training … she has done something called MERIT which is insulin… well diabetes training including insulin and non-insulin medications and she is currently trained to become a prescriber in diabetes being her sort of interest so there has definitely is a lot of overlap between their roles. And I think it is fair to say when she started there was a bit of a turf war going on. (laughs) (General practitioner 4*) [12]

“The GPs are hesitant to let go of the person because they like to give them more of a holistic approach and deal with all their conditions together. So, I think a lot of pharmacists are meeting resistance in that way.” (PIP 9)[6]

*Issues of interprofessional competition and professionals’ attempts to protect occupational jurisdiction in their work are well documented. Studies have revealed tensions between staff in relation to authority, legitimacy, expertise and efforts made to gain professional recognition in multidisciplinary working.* [13]

*Pharmacists feared that encroachment on some GP roles may be perceived as a threat to GPs in future. Participants recounted previous GP opposition to the approval of community pharmacists administering vaccinations in the country – however, it was acknowledged that this opposition may have been primarily linked to financial remuneration rather than role expansion. “I think there would definitely be resistance. Just from, I think nobody likes to feel like their…anything is being taken away from their role.” [Pharmacist 11] [14]*

**CMOC**

| **CMOC 9** | Unclear role boundaries and role definitions, leading to role overlap between professions (C) may lead to interprofessional tensions (O) because of professional rivalries (‘turf wars’) (M). |
| --- | --- |

**Theme 2- Personal characteristics**

**Exposure to stress, opportunities to learn, resilience**

***Exposure and resilience***

*‘I think ... stress ... can get yourself through difficult situations and I think some of those actually aid your learning, in really helpful ways. I think when it’s all the time and you wake up and dread the entire week, that’s not sustainable, that’s your burnout territory.’* [15]

*General exposure to and experience of different and challenging situations was a strong theme across groups, as this exchange illustrated:*

*‘And exposure to different situations.’ (FG2-06)*

*‘Yes, difficult situations, good situations. But I think it’s the more challenging situations that builds up the resilience.’ (FG2-08)*

*‘I would agree.’ (FG2-07) [16]*

**CMOC**

| **CMOC 10** | When practitioners have been exposed to, and successfully managed stressful situations / circumstances (C), they develop resilience (O) because these situations have provided them with opportunities to learn from (M) |
| --- | --- |

**Resilience, confidence, boundary articulation**

***Confidence to articulate boundaries***

*‘Thus, having the confidence to be assertive was raised as a feature of resilience, in relation to understanding one’s role and professional identity, and when dealing with patients and other health professionals:*

*‘Knowing what your role is and sticking to that, I suppose being assertive with other disciplines.’ (FG1-03) Knowing what your role is and sticking to that, I suppose being assertive with other disciplines.’ (FG1-03)* [16]

*This theme demonstrated an inter‐relationship whereby understanding scope and linking experience to confidence were very much attributes that meant that a clear understanding of responsibility could be understood. The quote below highlights the importance of recognition of scope and limits:*

*“Mark the boundaries where that responsibility stops”. (T3)*

*The quote below highlights a potentially vital component of the role. The relationship between experience and confidence was an area of interest, as all the clinicians were experienced practitioners who had several years of clinical experience to support their practice:*

*“It's difficult because with experience comes confidence”. (T4)[4]*

**CMOCs**

| **CMOC 11** | Practitioners who have greater experience of exposure to manageable stress and who possess greater resilience (C) have learnt where their boundaries are (M) allowing them to articulate their boundaries (O) |
| --- | --- |
| **CMOC 12** | When practitioners have had more exposure to and successfully managed more challenging patients (C) they are better able to understand where the boundaries of their practice are (O) because they have been able to learn from these experiences (M) |

**Training**

***Resilience training***

*‘Yes, there’s an element of that, if you’re not a resilient person, you can be taught skills that will stop you getting into a position where you then have to be a resilient person … like brief intervention skills, motivational interviewing, and all those kind of communication skills.’ (FG5-17) [1]*

*Developing resilience was considered to be possible through experience, learning from others, and formal training.* [16]

***Lack/ Insufficient training***

*This study highlighted a lack of consistency in training in relation to its content, supervision and mentorship with no assessment of competency. Without agreed standards of competency to measure against, there is a risk of inadequate training for those embarking on FCP roles [4]*

*Five (45%) participants stated that they felt there was a lack of appropriate training and mentorship available. These statements were more prevalent in the participants who were transitioning from band 6 to band 7 first contact physiotherapist roles [1]*

*‘Training was inadequate, we were left to learn on the job… [I felt] isolated and scared.[1]*

*The problem is there is no structured training at the moment, and I think there needs to be. There is definitely a need for it …. a structured portfolio or pathway definitely helps with competence and safer practice (PIP 11) [6]*

***Need for and helpfulness of training***

*Formal training is considered essential for the building of professional identity and status…however, across the studies there was variation in the training link workers received. While formal training could increase link worker confidence in performing their role, some link workers reported that it was often minimal, inconsistent and non-standardised. Some needed to draw on skills and training they received in previous roles[5]*

*The importance of ongoing professional training was recognised as nurturing health professionals’ resilience[16]*

*Participants who had received (ongoing/priori) formal education and training relevant to their PIP role mentioned how this had helped them develop the knowledge and skills necessary to adapt to their new roles and make an effective contribution to the practice, thus facilitating their integration.[6]*

*“This study has also highlighted the importance of training and support for pharmacists transitioning to work in general practice.”* [17]

*Dr B believed if a patient was to be seen by the advanced paramedic the doctors in the practice needed to be sure the paramedic was suitably qualified for this new advanced role. Dr B: . . . as a GP you are very protective of your skills and the amount of training you have had, but if you are going to have someone that is substituting you, you need to know . . . they have had good training.[18]*

***Need for training for complex primary care roles & beyond traditional professional boundaries***

*These more involved and longer-term approaches to social prescribing could be key in supporting individuals with multiple long-term conditions and experiences of socioeconomic deprivation. However, these more involved approaches may require additional time and training for link workers and may cause high levels of stress and emotional burden.[5]*

*The present work supports the evidence for enabling a programme of education and training for advanced practitioners and will therefore support any local or national programmes in the future [4]*

*Findings indicate that the GP setting requires a broad set of competencies—covering areas including the use of IT systems; clinical knowledge; and communicating with patients and other healthcare professionals. This implies that further emphasis on clinical and consultation skills should be added to training programmes aimed at GP pharmacists; furthermore, ongoing support is also needed with regards to generic skills such as the use of IT systems, documentation, and general procedures and processes within primary care, some of which might need to be tailored to the specific practice context [19]*

*“Future integration of pharmacists into general practice: improved training for pharmacists transitioning into general practice, setting clear expectations of the role from the outset.”[17]*

*Pharmacist*

*Among those participants who had received formal PIP training, some reported that this did not provide them with the full range of skills needed in a GP practice setting, particularly when required to provide consultations to patients with multiple conditions. “There’s a lot of things that could be related to blood pressure which could be easily done had you as a pharmacist had better physical, clinical training … I think that that’s a little bit of barrier because we do as pharmacists lack those clinical skills” (PIP 6) [6]*

*Link workers*

*For example, link workers who previously worked in mental health professions or had mental health training felt more confident and able to make decisions [5]*

*Physiotherapist*

*The training that they did receive provided a useful baseline; however, it lacked sufficient depth, with difficulties applying the theory to a first contact physiotherapy environment, where quick decisions, in a short time frame, were required*.[15]

*‘Therefore, training should include the management of non-MSK conditions, including mental health first aid and cultural competence training’* [20]

*‘I have had training on red flags obviously, I have had training on what to assess and what to think about but not specifically about managing uncertainty… it would be good for FCPs to have the opportunity to think about uncertainty, think about how they personally respond to it and deal and hold uncertainty’* (FCP 1) [21]

*Several of the FCPs spoke about diagnosis-centred uncertainty, which can affect the quality and safety of care and can elicit psychological responses, such as feelings of burnout described by several of the FCPs in this study and elsewhere. A lack of preparedness to manage mental ill health and issues of cultural competence were also highlighted as gaps in their knowledge. Therefore, training should include the management of non-MSK conditions, including mental health first aid and cultural competence training. [22]*

**CMOCs**

| **CMOC 13** | When adequate training, required for the complexity of novel primary care roles is not provided (C) practitioners experience high levels of stress (O) because they do not feel prepared for the roles (M) |
| --- | --- |
| **CMOC 14** | When practitioners new to primary care have been provided with adequate training to develop appropriate skills needed to manage challenges in their new role (C) they are more likely to cope better with the challenges they face (O) because they have the confidence to do so (M) |
| **CMOC 15** | If practitioners working as first contact practitioners have suitable training about how to deal with the diagnostic/ medical uncertainty related to the first contact consultation in general practice (C) they are more likely to cope better with the undifferentiated nature of the patient presentation (O) because they are more comfortable with uncertainty (M) |

**Experience**

**Experience, confidence, coping**

**Lack of experience, worry, stress**

***Experience and anxiety***

*Anxiety due to uncertainty and fear of bad outcome is most apparent in younger and less experienced staff.[22]*

*“Finally, burnout was perceived as a risk factor for first contact physiotherapists possessing limited advanced practice experience”* [1]

This gave a sense of naturally developing resilience with increasing maturity, experience of adversity, and familiarity with workplace expectations. This was expanded on in another group that linked it to confidence; *‘But there’s no doubt, through your career you become more resilient, you know. As a newly-qualified staff nurse or a newly qualified whatever, you know you’re much more vulnerable to pressures and what people say and how people react and you’re much more constrained by, you know “I haven’t managed to do this today”, and you feel terrible about it and all the rest of it. But as you get older … you are more mature, you do become more … yeah, more comfortable with yourself.’ (FG3-14 [16]*

Their confidence seemed to be influenced by their previous physiotherapy experience and role, with those appointed from lower bands (Band 6), generally feeling less confident: *“The rest of us, who have only worked in physio departments are almost starting from scratch really. I mean I’ve done a little bit [of screening tests for non-MSK conditions]”. P3.* With those previously employed at higher bands (Band 7 and above) feeling more confident in their FCP role:” *I think one thing that’s made life easier for myself is my career pathway. So, about a years’ worth of experience working in orthopaedics and interface clinics, at Band 8. So, it wasn’t like I was coming into the FCP role cold from a physio role. I think that a lot of my essential peer support actually came before I started”. P1. [20]*

*Mental health impacts [experienced by FCPs] included stress and anxiety and mental strain. Fear around missing red flags contributed to stress and anxiety. This FCP described this ‘worry’ and concern for less experienced FCPs: I think that's where my worry, sometimes, is that there are a lot of people within the role that don't have enough clinical experience to recognise that. And, probably why a lot of other FCPs get worried about missing red flags, and I think that can play on people from a stress point of view. (Grace)[23]*

*"Mentally, there are higher stress levels because you (FCP) are working within 15-minute appointment slots… to put students, who do not have the experience, in a highly timed and pressured environment with demanding patients would be difficult for them." [24]*

*Vigilance, confidence and experience are all attributes that give the practitioner and the patient the safety net that minimizes serious pathology being missed. This is balanced against knowing where the boundaries lie, thereby ensuring that patients for whom concern is a factor are always assessed by other clinicians with the skills and responsibility to evaluate the presentation further.[25]*

*[O]thers affirmed that multiple years of experience as a pharmacist in a clinical setting should be mandatory prior to integration into general practice. Nonetheless, the importance of having sufficient experience in a patient-facing setting was emphasised in order to perform better in the role. “Maybe a case of having, you know, a minimum two years' post-qualification experience in the community setting or something like that, or a clinical setting somewhere, would be beneficial. I think it would be…I think it might be…difficult, but not impossible, to do it straight out of college, because there's a lot of practical elements that you don't get.” [Pharmacist 12][14]*

**CMOCs**

| **CMOC 16** | When practitioners can draw on their previous experience, that is relevant to the challenges of complex primary care roles (C), they are more likely to cope better in the role (O) because they are more comfortable in their personal capabilities (M) |
| --- | --- |
| **CMOC 17** | When practitioners have limited relevant experience of what is required within these complex primary care roles (C) they will experience more stress and anxiety (O) because they are worried about doing the wrong thing (M) |
| **CMOC 18** | When practitioners are worried about doing the wrong thing due to limited relevant experience (C) they are greater risk of burnout (O) due to their feelings of stress/ anxiety (M) |

**Experience of exposure to conditions outside immediate professional scope of practice that are encountered in primary care roles, confident, coping**

***Broader, relevant experience***

*For example, link workers who previously worked in mental health professions or had mental health training felt more confident and able to make decisions [5]*

*I started to pick up this [pharmacological knowledge] when I started to work in the chronic pain management team. So, you might be in a clinic where you will see patients and advise it [medication] to the patients . . . . it is having that background knowledge about your quantitative indications [dosage] and about how these drugs interact. (Participant 4) [26]*

*Other aspects of relevant post-registration work experience were rotating PT posts across different clinical areas (e.g. cardiorespiratory and neurology). As the following interview account illustrates, the experience of clinical rotations in different core areas of PT were perceived as vital preparation for dealing with the complexities of patient presentations in musculoskeletal first contact physiotherapy. “For me, it was when I was working in the rotations and doing the core areas of physio. The knowledge of all those core areas, means you are not fazed when a patient presents with comorbidities”. (Participant 7) [26]*

***Exposure to issues, conditions outside clinical competence***

*Our study confirms findings from previous studies which show that PIPs face challenges with issues/queries on areas outside of their clinical competence. Pharmacist independent prescribers in the UK tend to be trained in a specific area, while their role in GP practice is quite broad and general, which limits their knowledge of prescribing outside their clinical area of competence.[6]*

*Among those participants who had received formal PIP training, some reported that this did not provide them with the full range of skills needed in a GP practice setting, particularly when required to provide consultations to patients with multiple conditions. “There’s a lot of things that could be related to blood pressure which could be easily done had you as a pharmacist had better physical, clinical training … I think that that’s a little bit of barrier because we do as pharmacists lack those clinical skills” (PIP 6)[6]*

*A total of five (45%) participants reported that they were often exposed to medical conditions, which required a broader knowledge base to identify that it was not a musculoskeletal issue. The impact of this exposure led some participants to lack confidence in managing patients independently, leading to heightened stress. [1]*

***Experience of advanced clinical practice***

*I think one thing that’s made life easier for myself is my career pathway. So, about a years’ worth of experience working in orthopaedics and interface clinics, at Band 8. So, it wasn’t like I was coming into the FCP role cold from a physio role. I think that a lot of my essential peer support actually came before I started. P1.[2]*

*Those from advanced practice backgrounds reported their prior experience helped but for some it was still not sufficient to prepare for the uncertainty of working in the primary care environment: I think theoretically I read everything about it, and I was very excited to do it but I would say zero percent ready. (FCP 2) [3]*

*The FCPs in this study felt confident in managing MSK conditions and demonstrated advanced skills in assessment, clinical reasoning, ordering and interpreting investigations, undertaking complex case management and making appropriate onward referrals [2]*

*burnout was an issue for physiotherapists in model 3 who completed four sessions a week in multiple surgeries, with minimal presence in each surgery, especially if they had lower levels of advanced practice. [1]*

*First Contact Practitioners participants reported confidence in the management of complexity within MSK cases utilising advanced skills but less confidence in managing non‐MSK conditions, particularly mental health. This was impacted by the experience and level of practice, with those in higher banded positions feeling more confident [27]*

**CMOC**

| **CMOC 19** | Where practitioners have had experience of exposure to conditions encountered within primary care roles that are outside their immediate professional scope of practice (C) they feel more able to cope (O) because they are more confident about what to do (M). |
| --- | --- |

**Theme 3- Complexity**

**Caseload complexity, burden of responsibility, stress**

***Caseload complexity***

‘*Participants highlighted that in their FCP role, they were dealing with more complex patients, which they had not anticipated at the outset’ [2]*

*FCPs were clear about the need to understand the medical elements of MSK practice in a first‐contact physiotherapy environment and the associated factors that can influence the clinical presentation: Well, comorbidities are massive, aren't they, like diabetes, cancers, neurological conditions, anything that's happened recently—falls, things that flag up frailty and things like that.* (T1) [4]

Skill mix implementation is challenging because of the inherent complexity of general practice caseloads [13]

***Caseload complexity, the increase in complexity because of unclear role boundaries and role definition, stress***

*However, this same lack of clear role definition could cause role stress through workload overload and complexity [5]*

*The lack of role clarity led to complexity, fragility and professional tensions due to overlap in responsibilities [12]*

*First Contact Practitioners participants reported confidence in the management of complexity within MSK cases utilising advanced skills but less confidence in managing non‐MSK conditions, particularly mental health. [27]*

***Unclear role boundaries and role definition***

*Example A*

*[A total of eight (73%) participants remarked that there was a lack of understanding of the first contact physiotherapist’s role…Some six (55%) participants also expressed that their patients did not understand the role…There was ambiguity concerning the purpose of the service, with several aims reported by the participants] [1]*

*A total of five (45%) participants reported that they were often exposed to medical conditions, which required a broader knowledge base to identify that it was not a musculoskeletal issue. The impact of this exposure led some participants to lack confidence in managing patients independently, leading to heightened stress.[1]*

*Example B*

*[However, there seemed to be some confusion about their role. This confusion was evident from patients, reception staff and GP…This loss of clarity in their role resulted in some FCPs frequently acting as second contact practitioners][2]*

*Most participants felt less confident in managing non-MSK conditions. This included non-MSK conditions masquerading as MSK conditions: It is all of the other stuff really; the assessment and treatment of kidney disease and the other masquerading things where I need to develop my knowledge. P6. [2]*

*Example C*

*[Some participants described situations, where PIPs had been asked to make decisions or carry out roles beyond their clinical competence due to GP practice teams’ lack of an understanding of their role.] [6]*

*"A lot of patients … will have polypharmacy, multi-morbidity and this is where the individual Pharmacist has to make their own decision as to what they’re happy to prescribe or not prescribe… I think that can cause problems because if the GPs see the Pharmacist as an Independent Prescriber… they may expect that Pharmacist to prescribe that medication but then individually the Pharmacist may feel that they’re not competent in that area.”* (PIP 5).[6]

***Caseload complexity due to psychosocial, socioeconomic and sociodemographic factors***

*Resilient individuals understood that the ability to be flexible and adaptable are essential…especially raised in both focus groups in rural locations. These health professionals may have to deal with unscheduled care for drop-in patients when there is no access to the patients’ medical histories. Therefore, rural health professionals must be prepared to deal with everything that turns up* [16]

*Some of the FCPs were working in practices with ethnically diverse populations. This presented with some challenges for the FCP in terms of consultation where the participant did not speak English and having to use a telephone translation service (language line): I had a week where I spoke to one person that had English as their first language; I’m going to guess out of 65 patients. Language line, by the time you’ve rang up, waited for the response, and then they’ve said unfortunately we don’t have that language. P4*. [2]

*Participants described additional challenges when working in areas of high socioeconomic deprivation including lack of access to technology, low digital literacy and communication barriers* [28]

*This meant that link workers needed to provide support for people with complex and/or severe mental health concerns, as well as support for those experiencing food poverty, unemployment, or needing benefits advice* [5]

*What I was quite surprised with is the amount of mental health issues coming through. I was like wow! There is a massive issue with that [2]*

Caseload complexity, the undifferentiated nature of the first contact consultation and consequences

*Nevertheless, the complex, unpredictable nature of this work was acknowledged as introducing some challenges and associated risk: “You don’t know what’s coming through the door. You can’t look at your list two weeks in advance and say, oh I’ve got X, Y and Z coming in then, let’s read up on it,it’s on the day you know, you have no prior warning” (FCP7). [29]*

*"There are challenges to working in an environment where the patient has not been screened by a GP, and this would commonly relate to safety concerns and the danger of missing ‘red flags.’".[4]*

*I am seeing stuff that would have gone straight to a rheumatology clinic; but now you’re the first person that is picking that up and doing the primary diagnosis. I’ve had a first diagnosis of multiple sclerosis in clinic; so quite a wide range of pathologies, but you need to have the clinical skills to recognise and know how to appropriately onward manage them. So, it’s being able to think on your feet quickly and making that decision there, so the patient is managed appropriately… it’s the level of clinical complexity that you’re dealing with. P1. [2]*

*This was particularly pertinent to the first contact nature of the role which involves direct access for patients without any prior triage by a GP or other healthcare professional. This lack of prior screening created additional risk amongst MSK FCPs, adding to the burden of responsibility: I feel there's a lot of responsibility and accountability in my role and a lot of that is generally down to the fact that we're seeing patients without them having been seen by anyone else before. (FCP 1) [3]*

"*You do not want to miss something [red flag] that would be horrible to miss. That weighs on us as FCPs, let alone for a student.".*[30]

*Fears of missing serious pathology masquerading as MSK pathology was also widespread. This accountability seemed to heavily influence uncertainty amongst the participants: It's the nasties, isn’t it? That is what worries me, the cancers and stuff like that. I have had one that turned out to be mets [metastatic cancer] rather than hip OA [osteoarthritis].* (FCP 7)[3]

*The uncertainty and worry related to missing serious pathology and the possible medico‐legal consequences was linked with potential over‐investigating: I think covering myself is a phrase that I have heard a lot as well because being in primary care, with the added risks, with the added uncertainty, I think I’m just more aware that there are medico‐legal cases that go on and… aware that my head is above the parapet a bit more.* (FCP 1)[3]

*The worry connected with uncertainty seemed too much for some who had decided to leave their role altogether: I think I have made the decision that I am going to look for something else, because you can't be going home every day worrying about patients and worrying about* *your job, you are going to end up having a breakdown. (FCP 7) [3]*

**CMOCs**

| **CMOC 20** | When practitioners have to manage patients with conditions that they are less familiar with managing (C) this can cause them stress (O) because they are not confident that they can manage these patients appropriately (M). |
| --- | --- |
| **CMOC 21** | When practitioners’ role boundaries and role definitions are unclear it adds to the conditions that practitioners are less familiar with managing, or they feel are outside their scope of practice (C) and this can cause them stress (O) because they are not confident that they can manage these patients appropriately (M). |
| **CMOC 22** | The diagnostic uncertainty associated with the undifferentiated patient that forms a fundamental part of the first contact role (C) causes practitioners to have turnover intentions (O) because they are worried about the consequences of making a mistake (M) |

**Nature of role, caseload complexity, practical and emotional consequences**

**The challenge of time**

*"Mentally, there are higher stress levels because you (FCP) are working within 15-minute appointment slots… to put students, who do not have the experience, in a highly timed and pressured environment with demanding patients would be difficult for them.".[30]*

*"Clinicians in the study generally reported having shorter appointment times in an FCP role than in their normal MSK physiotherapy roles. This meant that the pressure of time was a factor, and therefore the ability to recognize this as a challenge but to be able to make quick decisions was noted as a key theme within the data." [4]*

*that we are going to be really great at managing people’s health behaviours and we sit there and go ‘how are we going to do that, and differentiate what’s wrong with them, and give them initial treatment in 20 min?’ You can’t do it. P5.[2]*

*Participants highlighted that in their FCP role, they were dealing with more complex patients, which they had not anticipated at the outset: What I was quite surprised with is the amount of mental health issues coming through. I was like wow! There is a massive issue with that. I had one guy, who’s had osteoarthritis in his hips, just come in and break down, basically. He started crying and saying, “I can’t go on like this.” You know, having sort of suicidal thoughts. He’s lost his job. He’s not able to get out anymore. He’s really, really struggling. He’s on maximum opioids and the issue was he was morbidly obese, so he’s been turned down for surgery, so it was just like, whoa, I need to try and sort him out in that session somehow. P10. [2]*

*While more intensive and involved approaches to social prescribing may benefit disadvantaged groups, they require additional time, training, and support for link workers, and may not immediately deliver on social prescribing’s goal to reduce patient attendance in primary care [5]*

“*A total of six (55%) participants commented that they often overran within clinics, which was commonly related to patient presentations; five of these participants worked in service model 3. [1]*

*I was speaking to FCPs in [name of English region] and Wales, they were on 20 min and they were always overrunning by half an hour, or an hour, every day. Is that good for you to be doing that every day? I know the GPs do that and that is one of the main reasons we’re there, because they’re struggling to recruit GPs and, because of burnout, they’re going part-time. So, if we wanted it to be a full-time FCP, we’ve got to look after our health and wellbeing as well*. P10. [2]

*The communicative skill of letting the patient tell their story and allowing all concerns to be heard and addressed was a further key finding, and, when the time constraints of working in primary care are considered, then the ability to do this in such an environment demonstrates an important skill of the role.[4]*

***Practitioner strategies to manage uncertainty and mitigate risk.***

*Nevertheless, the complex, unpredictable nature of this work was acknowledged as introducing some challenges and associated risk: “You don’t know what’s coming through the door. You can’t look at your list two weeks in advance and say, oh I’ve got X, Y and Z coming in then, let’s read up on it,it’s on the day you know, you have no prior warning” (FCP7). Consequently, the FCPs spoke about the need to manage risk and associated competency requirements.[29]*

“*Diagnostic uncertainty existed with concerns around missing serious pathology and non-musculoskeletal conditions apparent. Possible over-utilisation of investigations and a negative impact on wellbeing were consequences associated with uncertainty”*[3]

“*Various strategies to help mitigate uncertainty were employed. Person-centred consultation approaches and the use of safety-netting were considered effective. Access to appropriate supervision and support networks across all employment models was seen as vital to aid both decision making and wellbeing*.”[3]

“*It comes down to safety netting, not just for the patient but for yourself. I mean we’ve all made errors in our careers, and you’ll never ever forget them, and I want to make sure that I’ve learnt from that. I know at some point something will come back and bite me; that’s worrying”.* P5.[2]

***Reaching out for support and sharing concerns***

*"A total of five (45%) participants reported that they were often exposed to medical conditions, which required a broader knowledge base to identify that it was not a musculoskeletal issue… In those instances, further advice from a physician was needed to order appropriate investigations and complete paperwork, which frequently led to overrunning. [1]*

*All the GP practices I work in there is always a GP there when we are working … I don’t feel anxious about being uncertain, I think because there are GPs nearby that I can ask if I am uncertain. P7*.[2]

*Support networks were reported to be both formal (clinical supervision and debrief sessions) and informal (messaging applications) in nature, with both considered a useful way to address uncertainty* [3]

*Well embedded workforce support and clinical supervision were key to being able to deal with the complex cases. Where organisational support for link workers was not in place, some could find themselves isolated which could lead to feelings of anxiety and decreased capacity to cope [5]*

*“[T]he vast majority (90%) saw it as important to share their uncertainty with patients and peers. Indeed, the majority suggested that sharing their uncertainty had the potential to improve their relationship with their patients”* [31]

*Anova revealed that both the anxiety due to uncertainty and the reluctance to disclose mistakes scores were significant. [22]*

**CMOCs**

| **CMOC 23** | When practitioners are not provided with the time they judge they need to appropriately manage a patient with complex problems (C), they may become distressed (O) because they feel they are not doing their job properly (M) |
| --- | --- |
| **CMOC 24** | When practitioners have to do more work to manage the caseload complexity and reduce diagnostic uncertainty that they experience in their role (C), these approaches usually result in clinics over-running (O) because these approaches often take up more time (M). |

**Theme 4- Work Environment**

## **Presence**

***Being co-located***

*All GPs reported they would like the pharmacists to work full-time, and would like to receive increased funding from the Health Board to accommodate this*.[17]

*Co-locating link workers in primary care, providing space for them in practices, was noted to help referrers to understand and remember the link worker role, as well as making the service seem trustworthy and credible to patients/service users [5]*

*The benefit of the job is that I’m now in the surgery and I can now provide some of that information to some of those people. For me that’s like wow that’s fantastic (…) some of the work GPs will come up with, “have you got any ideas about?” rather than actually refer people so for me the strength of the service is actually being visible, being there and the people who actually do the job are there able to give people quick access to, maybe sometimes quite basic information that they didn’t know exists.[9]*

*“When we have a multidisciplinary. . .team meeting. Talking about complex patients, we can include those first contact practitioners. They may not be doing what they would do if they were face to face to a patient. But they can provide help and support to the whole team about understanding those areas. For instance, we’re all learning about Start Back at the moment, they can train us in Start Back” Practice Manager 6. [29]*

*FCPs were seen as highly specialised MSK practitioners. These benefits were felt to be greater when the FCP service was collocated.[29]*

*There was no clear consensus regarding the hours that pharmacists should be available in the practice. It was acknowledged that the time may be constrained by funding and that the working hours per practice would depend on the workload requirements. Whilst having a full-time GPP role was viewed as a facilitator to integration, a part-time or sessional role may be deemed sufficient depending on the tasks that the GPPs would assume – which could then be expanded if required [14]*

*In this context, the use of organisational processes such as practice meetings or clinical meetings were perceived as being helpful to facilitate integration of PIPs. These multidisciplinary meetings provided PIPs with opportunities to demonstrate their knowledge and expertise by contributing to team discussions. “We get to know more about what’s happening in a practice and also, we can put our ideas forward as well. And feel more part of a team really.” (PIP13) [6]*

*The impact of effective interprofessional collaboration was discussed significantly more by six participants in models 1 and 2, where daily first contact physiotherapy cover was provided. Effective collaboration was thought to increase the knowledge base of the first contact physiotherapists, providing them with a greater awareness of medical conditions and the investigations required to diagnose them. This meant that healthcare efficiency was increased, and patients benefited.[1]*

*A strong preference was expressed for the pharmacy team to be located in house all day (GP6). In practices where the pharmacy team was located on-site, participants reported easy personal access and the ability to ask informal questions. Where the pharmacy team was located off-site, however, they were viewed as a separate entity (GP7) and aspects of communication were lost. Having somebody in house, (it) is the corridor talk and it’s difficult to quantify how helpful that is because you can say, “Can I just pick your brains on something?” If he wasn’t here, in the building, I don’t think I would think to ask him. Because he is in the building and because I see him… I do think “Oh actually we’ve got a pharmacist who can look into this” (GP3). [32]*

*(Being located off-site has) been more of a challenge because they (pharmacists) can’t just walk down to my office and say, “There’s a problem, can you help me?” Communication wise we have phones, our computer system, we can send instant messages, task, and email so it’s not an issue. Having said that, when you lose face-to-face contact, there could be more misunderstanding and delays (M5). [32]*

*Conflicting views were reported about working within more than one general practice as some perceived it …as a barrier as they thought that it lacks the continuity of practice and found it difficult to build new relationships with other healthcare professionals [11]*

*FCP was asked in the respondent validation whether he felt limited communication between GPs (across 25 Practices in the network) and the FCPs impacted on GPs' understanding, he responded: ‘Since we cover multiple Practices, the ‘home’ Practice has a pretty good understanding I think, and the other less so’. (FCP 3, Male) [33]*

*The majority of GPPs indicated that the most common method of communication between them and GPs was face-to-face [34]*

*Team-based care has been shown to enable doctors to work more collaboratively and reflexively. Evidence suggests that team-based care could improve continuity of care, with patients more likely to get the care they need. However, the contexts in which multidisciplinary teams operate are important and facilitating factors include co-location; a stable organisational structure; clearly defined roles and workflow; good communication through ‘huddles’, team meetings and informal ‘handoffs’ of patients; shared goals; and mutual respect and trust*. [13]

*Participants felt that co-location was an important facilitator in informal supervision, which would be less easily achieved through supervision carried out collaboratively with neighbouring practices. This could create difficulties for practitioners employed through the PCN: If they’ve got any concerns about a patient they talk to the doctors, they stop them in the corridor, they’re here so they stop them in the corridor and that’s the beauty of them being here. [ . . . ] it’s [location] massively important, it wouldn’t work if they weren’t based here, I can’t imagine working remotely with them, I really can’t. And that’s going to be a potential problem with the PCNs. ID18: site B, practice manager* [13]

**CMOCs**

| **CMOC 25** | When existing practice staff get more opportunities to interact with the ‘new’ practitioner (e.g. because they are visible and co-located in a practice) (C) this leads to more effective multiprofessional working (O) because staff get more chances to understand what each other can do (M) |
| --- | --- |
| **CMOC 26** | When practitioners are co-located in their new role (C), they find it easier to get and give support and help when they need it (O), because people are more physically accessible (corridor chats, staff meetings, MDT meetings, lunchtime) (M) |

**Isolation**

***Roles as isolating and consequences***

*In previous roles I’ve worked directly with my colleagues and bounced things off them. I’m finding I can’t do that. That can be hard, not having your work colleagues there to support you in ways you’re used to. P5.[2]*

*Feelings of isolation were discussed by the FCPs. Most of the FCPs felt isolated as they were not part of the practice team: You can feel quite isolated in your FCP role. Most of my career I’ve worked in a team and …we need support; emotional support. You know, it’s you in your four walls. The GPs they’re all very supportive, but they’re behind four walls as well. There’s very little interaction; there’s no meeting on a person-to-person level. Lunchtimes don’t overlap, so, you’re not getting to know these people. P8. [2]*

*This made some participants question whether they had made the right decision in taking the FCP post: If I was given the opportunity to go back two pay grades and be a standard physio, I’d take that opportunity right now. I feel like there’s no team ethos, I know we’re part of a team, but there’s no identity for us. I think emotionally the bad points outweigh the good points, but professionally the good points outweigh the bad points, so it’s a very tough place to be right now. P4. [2]*

*FCPs in primary care may be at risk of isolation and reduced learning opportunities.[35]*

*For two (18%) participants, mentorship was available within the surgeries in practice meetings where peer support occurred; however, this was not routine practice. The impact of inadequate training was that the transition from band 6 to band 7 role was difficult: ‘Training was inadequate, we were left to learn on the job… [I felt] isolated and scared.[1]*

*A total of four participants commented that the relationships they possessed within primary care lacked depth, with communication often via electronic notes or in corridor: ‘It was quite difficult to find the time to form those relationships as best as you could… It would be a hello and how are you doing in the corridors rather than maybe a discussion at lunchtime…’[1]*

*Recruitment and retention may be impacted by the risk of burnout due to isolation and high workload; this should be considered in role planning [27]*

*Moving to work in primary care was associated with feelings of isolation compared to traditional MSK physiotherapy departments: I think moving from a well‐knit, very socially accepted and accessible department feeling within physiotherapy outpatients to then being kind of a bit more isolated and out of the way. That's some of the reasoning behind some of my colleagues sidestepping away from FCP. (FCP 5)[3]*

*The feeling of isolation appeared interlinked with the ability to manage uncertainty. Clinical supervision offers an opportunity to discuss clinical cases or concerns, but access to this varied: I didn't have any supervision at all, zero, none. (FCP 8) [3]*

*Several participants described feeling isolated from other FCPs working as the sole therapists in primary care practice…It’s hard, you don’t get the same interaction [...] You can feel a bit isolated. [P07 T] [36]*

*Although this study focused on one health care professional group, potentially impeding the transferability of results to other groups, the findings illustrate key issues likely to be comparable to those of others working in isolation, such as general practice nurses, general practitioners, community pharmacists, or practice managers [36]*

***Proportionality to presence***

Less presence

*The impact of ineffective interprofessional collaboration was discussed exclusively by participants in model 3 (Ranged from two sessions a week (one session in the morning or afternoon) to one session every 2 weeks per GP surgery), with four commenting on it. A lack of knowledge of GPs’ treatment options was thought to lead to poorer patient outcomes, poorer quality first contact physiotherapy referrals and hindered the professional development of first contact physiotherapists. [1]*

More presence

*The impact of effective interprofessional collaboration was discussed significantly more by six participants in models 1 and 2 (presence every day), where daily first contact physiotherapy cover was provided. Effective collaboration was thought to increase the knowledge base of the first contact physiotherapists, providing them with a greater awareness of medical conditions and the investigations required to diagnose them. This meant that healthcare efficiency was increased, and patients benefited[1]*

*Practices that fully integrated the LWP had a better shared understanding of the programme, higher staff engagement, and implemented the LWP at all three of its intended levels of impact (patient, practice, and community).[37]*

***Modern day in working practice***

*Participants felt that co-location was an important facilitator… it’s [location] massively important, it wouldn’t work if they weren’t based here, I can’t imagine working remotely with them, I really can’t. And that’s going to be a potential problem with the PCNs. ID18: site B, practice manager[13]*

*Some staff expressed work dissatisfaction related to remote consultations in the form of frustration, isolation, lack of enjoyment, and increased workload. Frustration was raised as a result of patient difficulty describing symptoms or being asking to do inappropriate testing: I remember saying to someone over the telephone with a sprained ankle, can you stand on one foot. It belittles our profession; it totally belittles our profession. (Matt)[28]*

*Participants explained that ‘sitting in front of a screen all day’ was ‘very isolating’ and several FCPs experienced feeling separated from both their colleagues and patients. Some participants described missing the ‘fun’ and the ‘human’ contact of face‐to‐face consultations: I would find it very stressful.…there are things that I love about my job…. I wouldn’t be satisfied with my job if it was all digital. (Anna) [28]*

*GPs are spending more time in their rooms processing online consultations, increasing isolation and reducing informal interaction between staff and have larger workloads as a consequence of new administration tasks [28]*

***Approaches that combat isolation***

*Utilising Project ECHO for FCP CPD has the potential to reduce the isolation of clinicians working in Primary Care by creating a virtual community of practice, promoting knowledge exchange and improving clinician job satisfaction and patient care.[35]*

*One solution to this is portfolio roles whereby clinicians work across the MSK pathway to provide clinical variety and increase resilience (see Table 1). This also offers better integration of the role and transferrable skills which may improve recruitment and retention of staff whilst also enhancing the level of patient care through superior pathway knowledge. [38]*

*Being part of an established MSK workforce can provide a support network and having mentors for those new into advanced practice is crucial to help maintain resilience. [38]*

*The findings suggest that the Pharmacy Support and Development service at SEL GP Group is favourably perceived by stakeholders and thus far, has had a positive impact on integration and continued recruitment of pharmacy ARRS staff [39]*

*Findings revealed that virtual PLCs can be a valuable repository for providing networked learning opportunities and facilitating reflective practice amongst FCP physiotherapists.[40]*

*Twitter was indicated as a potential solution to overcome professional isolation, offering social networking with others to “promote conversation” (P09 T). Additionally, it was thought to provide an opportunity to learn, share best practices and access this study for evidence-based care often hidden behind journal paywalls [36]*

*The theme of secure base covered a range of factors related to link workers reporting that they had a safe base or team, which supported them to do their job or, alternatively, feeling isolated or that their security was under threat. I really feel so valued and I feel that sort of nurtured feeling that you get when you feel that people actually understand and listen and I often feel that the job that I do outside is reflected by what I feel when I get supervision, that sort of supervision and that attentiveness that people give me that I can then give to other people, for me that 110% yeah, [I] don’t recommend that anyone do this job without supervision it’s so valuable (…).[9]*

**CMOCs**

| **CMOC 27** | If practitioners don’t experience a team ethos that supports them in their new clinical setting (C) they feel isolated/ scared (O) because they don’t feel they belong (M) |
| --- | --- |
| **CMOC 28** | The isolation that practitioners experience when working in these novel primary care roles (C) may lead practitioners to consider leaving the role (O) because the role is not what they expected it to be (M) |
| **CMOC 29** | When there are systems in place to help practitioners overcome the challenges of isolating work practices (online consultations/ hub model) (C) it aids the development of practitioner resilience (O) because they feel more able to cope (M) |
| **CMOC 30** | When practitioners are made to work in ways that they believe are clinically inappropriate (C) they get frustrated (O) because they feel this is professionally compromising (M) |

**Capacity**

***Capacity less than demand***

*The stage 2 data collection reinforced these findings with the overriding consensus that there was currently insufficient capacity in FCP services to unburden GPs. For example, the site where 1 FCP provided 1 day of access for 20 GP practices: “I don’t think I’ve reduced the burden on GPs in terms of, I don’t think I’ve increased their capacity, purely because I’m doing two sessions for 22 GP practices, so I don’t think they’ll notice a difference” FCP 4* [29]

*It was also acknowledged by participants that the current funding models were seen as insufficient to realise the capacity increase required to genuinely impact GP workload to a measurable level. This is a pertinent finding with the current nationwide commitment to FCP whereby a population f 50,000 receives funding for 1 full time equivalent FCP. [29]*

*This study identified that an insufficient number of FCPs could result in an increase in waiting times [33]*

*So, I’ve got a 6-week waiting list, which as a FCP, shouldn’t exist, I’m not here in a physio role. And I don’t think they get that. P2 [2]*

*Two of the participants felt that the waiting lists had developed because musculoskeletal demand outweighed capacity, preventing the participants working as the first contact: ‘So because we were in the GP practices so infrequently, that next appointment slot might be 3 or 4 weeks down the line, and they [GPs] would just book it [after having seen the patient], but then the patient was waiting to see us, which stopped it being first contact and started it just being a physio waiting list.’ [1]*

*Some GPs referenced the ability to increase consultation times due to increased capacity; this was seen as a positive step. However, the level of service provision was not sufficient in all areas to determine these benefits. [27]*

***Other features impacting clinical capacity***

***Appointment time***

*The CSP [UK physiotherapy profession body] seem to really push that we are going to be really great at managing people’s health behaviours and we sit there and go ‘how are we going to do that, and differentiate what’s wrong with them, and give them initial treatment in 20 min?’ You can’t do it. P5. Twenty minutes is tight. I know that my colleagues do it in 20 min, but what’s the quality we’re giving patients. But, obviously, I understand from a cost and financial point of view that might be the way in needs to go. P10.[2]*

*Participants' (n = 13) suggested that the sheer pace and complexity of MSK FCP's work in time-limited appointment slots with potentially difficult patients could ultimately lead to student burnout. ‘… mentally, there are higher stress levels, because you (FCP) are working within 15 minute appointment slots … to put students, who do not have the experience in a highly timed and pressured environment and with demanding patients would be difficult for them’. (9) [30]*

*Time pressures were considered one of the most common factors contributing to uncertainty in the primary care environment: The volume, the pace is very different. I was used to 40‐minute appointments in a musculoskeletal assessment team… I think when you've got a 20‐minute appointment you have to really streamline a lot of your assessment process. (FCP 4)[3]*

*time pressures, it was also felt to be associated with a risk for patient safety: If you're time constrained, and your behind and you've got letters and scans and bits and pieces to do actually are you given that next patient your full attention? Or are you so wrapped up in all the other pressures and things that you've got to do that sort of drop the ball for a second. (FCP 3) [3]*

*Face-to-face appointment times ranged from 15 to 30 minutes; most lasted 20 minutes (71%, 50/70). Although not asked directly, some respondents indicated planned reductions in appointment times: two from 30-minuteappointments to 20-minutes and another indicated dissatisfaction with pressure from their practice manager to reduce from 20-minute to 10-minute slots.[41]*

*Time pressures had a big impact on professional resilience, including limited patient appointment times and the view that workers need sufficient breaks during the day to maintain concentration: ‘I suppose patients coming in with multiple problems can stretch your resilience because you think you know, you’ve got your time, your appointment time to deal with.’ (FG3-13) [16]*

*However, these more involved approaches may require additional time and training for link workers, and may cause high levels of stress and emotional burden [5]*

***Admin tasks***

*Thirty-one respondents (44%) reported administration time within their FCP role (range15–80 minutes), although some described time per session, per day or per number of appointments and others indicated availability of a non-specified time for administration [41]*

*The analysis showed those individuals who have between 0 and 9 non‐clinical hours per month and are male were in the ‘at risk’ category of burnout as per the BAT scoring system.[42]*

*Non‐clinical time has a direct influence on reducing burn out in clinicians and more effort is needed by employers to increase or implement this into FCP roles to protect the longevity and sustainability of the role in primary care. [42]*

*The issue of workload including information overload and volumes of paperwork while managing the practicalities of patient care was raised in all groups: ‘There’s so much paperwork in the health service across all disciplines.’ (FG4-16) [16]*

*Completion of the patient recorded outcome measures required for payment through the SIB also shaped work routines. The combined effect was to obstruct some routines, such as face to face meetings, whilst encouraging others, such as telephoning clients to secure referrals and complete PROMs. These routines contributed to a high staff turnover. [43]*

*Other issues were highlighted relating to supporting infrastructure such as differences in the NHS IT systems: The CCG operate from 3 different systems with different passwords to get on the computers. So, I’ve got six different passwords to get into the 7 clinics. Then I’ve got another six passwords to get onto their site specific software. So, I’ve got a diary full of passwords, and it takes me 15 min to get on the system! P4.[2]*

*NHS governance was also highlighted as impacting their role: For example, still to this day, I can’t order an x-ray. And we’re getting to the point now, where I’m feeling it may affect patient safety. P2. This led to frustration in their FCP role: It’s like boxing with no hands. We haven’t got the tools to do the job, the CCG promised us bloods, they promised us. They promised us that we would be getting pulse oximeters and blood pressure monitors, because they want us to take that information on each assessment as a first contact. We haven’t got the tools to do the job, we haven’t got the education to do the job, currently we’re doing a job that we are struggling to do. P4. [2]*

*However, not all FCPs seemed to be having these issues and felt the NHS systems were working well for them: The other good things, is having access to all the information. Having the GP records, for example, we’ve got all the old x-ray results within the GP practice. It’s just one click of a button and it’s there and we can get on with it straight away because you’ve got that information. P3. [2]*

***System-wide factors impacting clinical capacity***

*The lived experience of link workers, as depicted in papers included in this review, highlights how the state of the wider health and care system both impacts their workloads and their role stress due to the fact that they may find themselves “holding” service users who they cannot connect onwards due to relevant services or support either not being present or having limited capacity [5]*

*Respondents reported that a combination of this lack of understanding of the role and long waits for MSK/outpatients Physiotherapy services often led to FCPP appointments being used as a ‘fast‐track’ to Physiotherapy or as ‘in‐house’ Physiotherapy, n = 14 (16%). This also seemed to contribute to duplicate appointments, which 13 respondents (15%) reported as a problem, as two respondents explained ‘Often booked in after GP appointments, duplicating the appointment and sometimes attempting to bypass MSK waiting times. Some GPs certainly view us as fast access physio and misinform patient that they will have a course of physio’*. [8]

*It was acknowledged that the time may be constrained by funding and that the working hours per practice would depend on the workload requirements [14]*

*Organisational targets and funding models can shape a link worker’s role, sometimes leading to a focus on quantity of referrals rather than person-centred approaches [5]*

*It appears that the lack of mentorship may relate to funding: ‘Unfortunately, the practices I work at have said no to mentorship. Initially, they said one of the GPs agreed to do it and then they said, because they don’t get funding for that GPs time to do the mentorship, they’re not willing to do it. P10. [2]*

*From a business perspective, concerns were identified about the reduction in consultation length from 30 to 20 min, and several FCPs were advised to stop joint and soft tissue injections. Both were viewed as relating to cost [27]*

*Several FCPs highlighted how a change in funding had impacted their role, whereby restrictions to undertaking FCP injections had been implemented due to GP practices only being paid if the intervention was carried out by the GP and not by the FCP: I have been asked to stop injecting and I’ve been told the reason behind that is the funding. P8.[2]*

*Additionally, a change in Primary Care Network (PCN) funding led to further anxiety relating to the future of FCP in different localities. There was much uncertainty voiced and concern raised whether the FCP role was affordable: The only thing that would be a negative on the horizon is what’s happing, in terms of PCNs and the contracts. I don’t think they can afford to keep their own staff on because it’s such a big practice. P8.[2]*

*All GPs reported they would like the pharmacists to work full-time, and would like to receive increased funding from the Health Board to accommodate this [17]*

**CMOC**

| **CMOC 31** | When services are set up in ways that make it challenging for the practitioner to carry out what they think is expected of them (C), they get frustrated (O) because they feel they are being asked to do the impossible (M) |
| --- | --- |

**Supervision**

***Supervision***

*Participants felt that co-location was an important facilitator in informal supervision, which would be less easily achieved through supervision carried out collaboratively with neighbouring practices. This could create difficulties for practitioners employed through the PCN: If they’ve got any concerns about a patient they talk to the doctors, they stop them in the corridor, they’re here so they stop them in the corridor and that’s the beauty of them being here. [ . . . ] it’s [location] massively important, it wouldn’t work if they weren’t based here, I can’t imagine working remotely with them, I really can’t. And that’s going to be a potential problem with the PCNs. ID18: site B, practice manager [13]*

*Informal supervision and support also took place during daily coffee/lunch breaks, allowing people to get advice and share an understanding of guidelines and best practice. Shared breaks also strengthened horizontal relationships and openness between staff members, which facilitated informal advice-seeking. [13]*

*The theme of secure base covered a range of factors related to linkworkers reporting that they had a safe base or team, which supported them to do their job or, alternatively, feeling isolated or that their security was under threat. I really feel so valued and I feel that sort of nurtured feeling that you get when you feel that people actually understand and listen and I often feel that the job that I do outside is reflected by what I feel when I get supervision, that sort of supervision and that attentiveness that people give me that I can then give to other people, for me that 110% yeah, [I] don’t recommend that anyone do this job without supervision it’s so valuable (…).[9]*

*The importance of debriefing and provision of clinical supervision is strongly emphasised in the capability roadmap produced for MSK FCPs in England. Not being able to debrief or access supervision may perpetuate any uncertainty for MSK FCPs and lead to worry impacting on psychological wellbeing [3]*

*The linkworkers’ prophesies of the erosion of protective factors turned out to be accurate. Informal team contact, which promoted good working relationships and enabled ideas and solutions to be shared reduced over time as work pressures increased. It is doubtful that losing this team time increased efficiency or effectiveness. The containment and opportunities for reflection provided by supervision also seemed less evident. A positive culture of mutual respect and shared vision takes time to develop and can be all too easily lost in responding to changes without time to reflect on their impact. In this research, one of the effects of this loss seemed to be disillusionment [9]*

*Formal and informal supervision covered various activities and provided supervising GPs with insights into how practitioners work and their level of skill [13]*

*Well embedded workforce support and clinical supervision were key to being able to deal with the complex cases [5]*

*"The introduction of several of these new roles requires substantial supervision from GPs and complex changes to how work is organised that may ultimately reduce efficiency.". [44]*

*Support networks were reported to be both formal (clinical supervision and debrief sessions) and informal (messaging applications) in nature, with both considered a useful way to address uncertainty: So those daily debrief sessions or any kind of supervision sessions, I think they help and they are really, really important. (FCP 2) It's a hot channel, the WhatsApp! I have had to mute it a few times. But you know that has been really helpful. (FCP 4)[3]*

*Access to appropriate supervision and support networks across all employment models was seen as vital to aid both decision making and wellbeing.[3]*

*This study supports the recent CSP (2020a) document in which it states sufficient time should be allocated for CPD, training and supervision. [42]*

**CMOCs**

| **CMOC 32** | When suitable supervision is provided to practitioners in ways that they can access (C) they feel more able to undertake their role (O) because they feel supported (M) |
| --- | --- |
| **CMOC 33** | Co-location provides greater potential access to both formal and informal supervision (C) which supports practitioners in their roles (O) as it provides them with the support, they feel they need (M) |

[1] Lewis MW, Gill P. Facilitators and barriers regarding the implementation and interprofessional collaboration of a first contact physiotherapy service in primary care in Wales: A qualitative study. Int J Ther Rehabil 2023;30. https://doi.org/10.12968/ijtr.2022.0053.

[2] Greenhalgh S, Selfe J, Yeowell G. A qualitative study to explore the experiences of first contact physiotherapy practitioners in the NHS and their experiences of their first contact role. Musculoskelet Sci Pract 2020;50. https://doi.org/10.1016/j.msksp.2020.102267.

[3] Ingram S, Stenner R, May S. The experiences of uncertainty amongst musculoskeletal physiotherapists in first contact practitioner roles within primary care. Musculoskeletal Care 2023;21:644–54. https://doi.org/10.1002/msc.1735.

[4] Langridge N. The skills, knowledge and attributes needed as a first-contact physiotherapist in musculoskeletal healthcare. Musculoskeletal Care 2019;17:253–60. https://doi.org/10.1002/msc.1401.

[5] Turk A, Tierney S, Hogan B, Mahtani KR, Pope C. A meta-ethnography of the factors that shape link workers’ experiences of social prescribing. BMC Med 2024;22. https://doi.org/10.1186/s12916-024-03478-w.

[6] Alshehri AA, Hindi AMK, Cheema E, Sayeed Haque M, Jalal Z, Yahyouche A. Integration of pharmacist independent prescribers into general practice: a mixed-methods study of pharmacists’ and patients’ views. J Pharm Policy Pract 2023;16. https://doi.org/10.1186/s40545-023-00520-9.

[7] Maskrey M, Johnson CF, Cormack J, Ryan M, Macdonald H. Releasing GP capacity with pharmacy prescribing support and New Ways of Working: A prospective observational cohort study. British Journal of General Practice 2018;68:e735–42. https://doi.org/10.3399/bjgp18X699137.

[8] Lamb K, Comer C, Walsh N, McHugh G. Patient access to first contact practitioner physiotherapists in the UK: A national survey. Musculoskeletal Care 2023;21:1554–62. https://doi.org/10.1002/msc.1834.

[9] Bowden GE, Smith JCE, Parker PA, Boxall MJC. Working on the Edge: Stresses and Rewards of Work in a Front-line Mental Health Service. Clin Psychol Psychother 2015;22:488–501. https://doi.org/10.1002/cpp.1912.

[10] Bartlett S. Practice-based pharmacists: considerations for general practices. British Journal of General Practice 2023;73:249–50. https://doi.org/10.3399/bjgp23X732909.

[11] S. Alghamdi RD and KH. Independent pharmacist prescribers’ views of their role as prescribers in primary care settings in Wales. International Journal of Pharmacy Practice 2020;28:4–43. https://doi.org/10.1111/ijpp.12606.

[12] Nabhani‐Gebara S, Fletcher S, Shamim A, May L, Butt N, Chagger S, et al. General practice pharmacists in England: Integration, mediation and professional dynamics. Research in Social and Administrative Pharmacy 2020;16:17–24. https://doi.org/10.1016/j.sapharm.2019.01.014.

[13] McDermott I, Spooner S, Goff M, Gibson J, Dalgarno E, Francetic I, et al. Scale, scope and impact of skill mix change in primary care in England: a mixed-methods study. Health and Social Care Delivery Research 2022;10. https://doi.org/10.3310/YWTU6690.

[14] Morcos P, Dalton K. Exploring pharmacists’ perceptions of integrating pharmacists into the general practice setting. Exploratory Research in Clinical and Social Pharmacy 2021;2. https://doi.org/10.1016/j.rcsop.2021.100027.

[15] Lewis MW, Gill P. Facilitators and barriers regarding the implementation and interprofessional collaboration of a first contact physiotherapy service in primary care in Wales: A qualitative study. Int J Ther Rehabil 2023;30. https://doi.org/10.12968/ijtr.2022.0053.

[16] Matheson C, Robertson HD, Elliott AM, Iversen L, Murchie P. Resilience of primary healthcare professionals working in challenging environments: A focus group study. British Journal of General Practice 2016;66:e507–15. https://doi.org/10.3399/bjgp16X685285.

[17] A. Iqbal and M. Allinson. Evaluation of pharmacists working in a GP practice in South Wales. British Journal of General Practice 2013;63. https://doi.org/10.3399/bjgp13X669194.

[18] Muldoon D, Seenan C. The introduction of advanced paramedics into primary care in Northern Ireland: a qualitative descriptive study of the experiences of general practitioners. Br Paramed J 2021;6:1–6. https://doi.org/10.29045/14784726.2021.12.6.3.1.

[19] Mueller T, Preston KE, Mcfadyen Weir N, Bennie M, Newham R. Competencies required for General Practice Clinical Pharmacists providing the Scottish Pharmacotherapy Service: A modified eDelphi study. Health Soc Care Community 2021;29:e328–37. https://doi.org/10.1111/hsc.13357.

[20] Greenhalgh S, Selfe J, Yeowell G. A qualitative study to explore the experiences of first contact physiotherapy practitioners in the NHS and their experiences of their first contact role. Musculoskelet Sci Pract 2020;50. https://doi.org/10.1016/j.msksp.2020.102267.

[21] Ingram S, Stenner R, May S. The experiences of uncertainty amongst musculoskeletal physiotherapists in first contact practitioner roles within primary care. Musculoskeletal Care 2023;21:644–54. https://doi.org/10.1002/msc.1735.

[22] Ashton L. Does medical uncertainty affect physiotherapist practitioners working within a ﬁrst contact role? An exploratory study. Physiotherapy 2020;107:e158–9. https://doi.org/10.1016/j.physio.2020.03.231.

[23] Anchors Z, Jones B, Thomas R, Berry A, Walsh N. The impact of remote consultations on the health and wellbeing of first contact physiotherapists in primary care: A mixed methods study. Musculoskeletal Care 2023;21:655–66. https://doi.org/10.1002/msc.1737.

[24] Bassett AM, Jackson J. Challenges and Learning Opportunities of Pre-Registration Physiotherapy Placements in First Contact Settings: The Perspectives of Musculoskeletal First Contact Physiotherapists. Musculoskeletal Care 2020;18:140–9. https://doi.org/10.1002/msc.1446.

[25] Langridge N. The skills, knowledge and attributes needed as a first-contact physiotherapist in musculoskeletal healthcare. Musculoskeletal Care 2019;17:253–60. https://doi.org/10.1002/msc.1401.

[26] Bassett AM, Jackson J. The professional development and career journey into musculoskeletal first contact physiotherapy: a telephone interview study. Physiother Theory Pract 2022;38:1453–68. https://doi.org/10.1080/09593985.2021.1872127.

[27] Bicker G, Hadley-Barrows T, Saunders A, Mairs H, Stevenson K. A narrative synthesis of the effectiveness and acceptability of musculoskeletal first contact physiotherapy practitioner roles in primary care. Musculoskeletal Care 2024;22. https://doi.org/10.1002/msc.1875.

[28] Anchors Z, Jones B, Thomas R, Berry A, Walsh N. The impact of remote consultations on the health and wellbeing of first contact physiotherapists in primary care: A mixed methods study. Musculoskeletal Care 2023;21:655–66. https://doi.org/10.1002/msc.1737.

[29] Goodwin R, Moffatt F, Hendrick P, Stynes S, Bishop A, Logan P. Evaluation of the First Contact Physiotherapy (FCP) model of primary care: a qualitative insight. Physiotherapy (United Kingdom) 2021;113:209–16. https://doi.org/10.1016/j.physio.2021.08.003.

[30] Bassett AM, Jackson J. Challenges and Learning Opportunities of Pre-Registration Physiotherapy Placements in First Contact Settings: The Perspectives of Musculoskeletal First Contact Physiotherapists. Musculoskeletal Care 2020;18:140–9. https://doi.org/10.1002/msc.1446.

[31] Goodwin R. HP, MF. Dealing with uncertainty as a first contact practitioner- a mixed methods evaluation. Physiotherapy 2024;123:e114–5. https://doi.org/10.1016/j.physio.2024.04.142.

[32] Ryan K, Patel N, Lau WM, Abu-Elmagd H, Stretch G, Pinney H. Pharmacists in general practice: A qualitative interview case study of stakeholders’ experiences in a West London GP federation. BMC Health Serv Res 2018;18. https://doi.org/10.1186/s12913-018-3056-3.

[33] Morris L, Moule P, Pearson J, Foster D, Walsh N. Patient acceptability of the physiotherapy first contact practitioner role in primary care: A realist informed qualitative study. Musculoskeletal Care 2021;19:38–51. https://doi.org/10.1002/msc.1505.

[34] A. H. F. Hassan HEB and CMH. An exploration of general practice pharmacists’ (GPPs) views on their role in general practice a cross-sectional questionnaire study 2023.

[35] Manson J, Jackson H, Setchfield I. Enhancing first contact practitioners’ continuous professional development within an integrated care system using project ECHO (Extension for Community Health Outcomes). Physiotherapy 2022;114:e89. https://doi.org/10.1016/j.physio.2021.12.031.

[36] Campbell L, Quicke J, Stevenson K, Paskins Z, Dziedzic K, Swaithes L. Using Twitter (X) to Mobilize Knowledge for First Contact Physiotherapists: Qualitative Study. J Med Internet Res 2024;26. https://doi.org/10.2196/55680.

[37] Chng NR, Hawkins K, Fitzpatrick B, O’Donnell CA, Mackenzie M, Wyke S, et al. Implementing social prescribing in primary care in areas of high socioeconomic deprivation: Process evaluation of the “Deep End” community Links Worker Programme. British Journal of General Practice 2021;71:E912–20. https://doi.org/10.3399/BJGP.2020.1153.

[38] Ingram S, Stenner R, Acton T, Armitage K. Implementation of a provider based musculoskeletal first contact physiotherapy service model: Key points to consider. Musculoskeletal Care 2021;19:232–5. https://doi.org/10.1002/msc.1527.

[39] Mohamed H, Alldred S. A service evaluation and stakeholder perspectives of the “Pharmacy Support and Development” Service across a GP Federation. vol. 31. 2023.

[40] Williams R. THE VALUE OF VIRTUAL PROFESSIONAL LEARNING COMMUNITIES TO SUPPORT CLINICAL EDUCATION AND TELEHEALTH PRACTICE. Physiotherapy 2024;123:e75–6. https://doi.org/10.1016/j.physio.2024.04.091.

[41] Halls S, Thomas R, Stott H, Cupples ME, Kersten P, Cramp F, et al. Provision of first contact physiotherapy in primary care across the UK: a survey of the service. Physiotherapy (United Kingdom) 2020;108:2–9. https://doi.org/10.1016/j.physio.2020.04.005.

[42] Nozedar L, O’Shea S. What is the prevalence of burnout amongst first contact physiotherapists working within primary care? Musculoskeletal Care 2023;21:776–85. https://doi.org/10.1002/msc.1752.

[43] Griffith B, Moffatt S, Pollard T. Link working at the intersections: an ethnographic exploration of delivering social prescribing in primary care. J Epidemiol Community Health, BMJ; 2021, p. A42.1-A42. https://doi.org/10.1136/jech-2021-ssmabstracts.88.

[44] Francetic I, Gibson J, Spooner S, Checkland K, Sutton M. Skill-mix change and outcomes in primary care: Longitudinal analysis of general practices in England 2015–2019. Soc Sci Med 2022;308. https://doi.org/10.1016/j.socscimed.2022.115224.
